# In Dogs We Trust? Intersubjectivity, Response-Able Relations, and the Making of Mine Detector Dogs

**DOI:** 10.1002/jhbs.21642

**Published:** 2013-12-06

**Authors:** Robert G W Kirk

**Affiliations:** Wellcome Trust Research Fellow based in the Centre for the History of Science, Technology, and Medicine at the University of Manchester(UK)

## Abstract

The utility of the dog as a mine detector has divided the mine clearance community since dogs were first used for this purpose during the Second World War. This paper adopts a historical perspective to investigate how, why, and to what consequence, the use of minedogs remains contested despite decades of research into their abilities. It explores the changing factors that have made it possible to think that dogs could, or could not, serve as reliable detectors of landmines over time. Beginning with an analysis of the wartime context that shaped the creation of minedogs, the paper then examines two contemporaneous investigations undertaken in the 1950s. The first, a British investigation pursued by the anatomist Solly Zuckerman, concluded that dogs could never be the mine hunter's best friend. The second, an American study led by the parapsychologist J. B. Rhine, suggested dogs were potentially useful for mine clearance. Drawing on literature from science studies and the emerging subdiscipline of “animal studies,” it is argued that cross-species intersubjectivity played a significant role in determining these different positions. The conceptual landscapes of Zuckerman and Rhine's disciplinary backgrounds are shown to have produced distinct approaches to managing cross-species relations, thus explaining how diverse opinions on minedog can coexist. In conclusion, it is shown that the way one structures relationships between humans and animals has profound impact on the knowledge and labor subsequently produced, a process that cannot be separated from ethical consequence.

“learning how to address the creatures being studied is not the result of scientific theoretical understanding, it is the condition of this understanding”

Vinciance Despret (2004, p. 131).

On September 3, 1982, Solly Zuckerman publically rebuked the use of dogs to locate landmines left by Argentinean forces during the recently concluded Falklands War. Citing extensive experimental investigations carried out on behalf of the Ministry of Defence in the 1950s, which Zuckerman had personally overseen, he asserted that “dogs can never be the mine hunter's best friend” (Zuckerman, 1982). In spite of Zuckerman's position as perhaps the most politically prominent scientist of mid-to-late twentieth- century Britain, not everybody agreed with this claim. Peter Norbury, who had commanded one of the first British Army Minedog Platoons between 1944 and 1945, affirmed that dogs were remarkably adept at using their acute “sense of smell” alongside a “highly developed sixth sense” to successfully detect buried landmines (see Figure 1). Speaking from personal experience, Norbury rejected “in the strongest of terms Lord Zuckerman's assertion that finding mines is beyond the capacity of a dog.” On the contrary, Norbury explained, dogs “consistently found mines in very varied terrain and in all sorts of conditions” and, should there be any further doubt, he offered to present his still intact “feet to prove it” (Norbury, 1982). This public disagreement illustrates two poles of a debate that had remained vigorously contested since dogs were first employed to detect landmines during the Second World War. Mine clearance risks lives as a matter of routine. Knowing whether dogs can or cannot be trusted in this role is therefore critically important. Nevertheless, despite decades of investigation, the capacity of dogs to detect landmines continues to divide the international mine clearance community.

Uncertainty over the utility of minedogs is not for want of need, will, or effort in seeking an answer. Antipersonnel mines and antitank mines are a significant military and civilian problem across the world. Accordingly, landmine clearance has acquired a prominent public and political profile, transitioning as a result from a predominantly military to a humanitarian concern. In 1997, for instance, the *Convention on the Prohibition of the Use, Stockpiling, Production and Transfer of Anti-Personnel Mines and on their Destruction* (1997) committed signatory nations to the active removal of existing landmines, while simultaneously prohibiting their future use. However, despite considerable efforts, there has been little progress in developing reliable technologies for detecting and disarming nonmetallic landmines (which pose unique problems since they evade metal detecting technologies). In the absence of a (mechanical) technological solution, faith in “Mine Detector Dogs,” one of the earliest responses to the challenge of nonmetallic mines, continues. The use of dogs was, and is, based on the assumption that they possess superior sensory capacities. Following a similar logic, the twenty-first century has witnessed alternative attempts to enroll nonhuman animals into this work. One of the most prominent are so-called “HeroRATS,” African (Gambian) pouched rats trained to detect landmines by Apopo, a Tanzania-based Belgian company.^1^ The U.S. Defence Advanced Research Projects Agency (DARPA) has alternated throughout its existence between attempts to develop a reliable mechanical “artificial nose” and, when this fails, a search for a nonhuman species that could be successfully employed to detect buried landmines. Most recently, DARPA sponsored Jerry J. Bromenshenk's attempts to utilize bees for this purpose (Bromenshenk et al., 2003). These and other innovations are mirrored by continued attempts to prove (or improve depending on one's position) the utility of minedogs by better harnessing their sensory capabilities to serve the humanitarian work of mine clearance.

This article does not attempt to answer the question as to whether dogs should, or should not, be trusted in the role of “mine detector.” Rather, a historical perspective is adopted in order to examine the factors that have made it possible to think that dogs can, or cannot, reliably detect buried landmine. At the heart of this debate is the question of how one can know what an animal can and cannot do. The main argument advanced by this article is that the capacities of minedogs remain both contentious and undecided because the answer to the question of what dogs can or cannot do is dependent on the way one relates to the dog in question. The way one addresses an animal shapes subsequent beliefs about the work that one can do with them. Put another way, different interconnected epistemological assumptions about how one can know a dog have led to different conclusions about the work one can do with dogs and thus their value as mine detectors. More specifically, an openness to work with, as opposed to exclude, cross-species intersubjectivity, has been the key point of difference between those who trust dogs to detect landmines and those who do not.

Conventionally, intersubjectivity refers to the often implicit shared space between conscious (subjective) human minds. Intersubjectivity brings to the fore processes of communal meaning-making and shared becoming by emphasizing consensus, codevelopment, and coproduction. Recently, some scholars have begun to extend intersubjective analysis beyond the human. The bioanthropologist Barbara Smuts, for instance, has mapped cross-species intersubjectivity across a hierarchical scale in an attempt to capture the variety of intersubjective relations that might be shared between human and nonhuman animals (Smuts, 2001). Significantly, heightened intersubjectivity has been connected to empathic relations (e.g., Thompson, 2001). Donna Haraway, for example, has identified cross-species intersubjectivity as a space in which one can develop a “response-able” relationship, within which “always more than one responsive entity is in the process of becoming.” According to Haraway, relating response-ably requires constant attentiveness to the “inside connections that demand and enable response,” a task that remains obligatory regardless of the species concerned (Haraway, 2008, p. 71). One important consequence of the intersubjective turn is that it obliges us to consider living beings as multiples, shaped through their on-going dynamic relations to others. Consequently, Haraway can write that to relate response-ably always already has ethical consequence because the past, present, and future of living beings is made and remade through their shared relations. This approach adapts the work of Vinciane Despret (2004), who has described this process as one of “becoming with” and advocates its study as a means to better understand understandings of the work that can be done across species. Collectively, this and related work emerging from the interdisciplinary field of “Animal Studies,” suggests that the questions asked of nonhuman animals, and the ways in which questions are asked, critically shape the possible answers animals can provide (always already with ethical consequence). This perspective is both a consequence of, and has consequence for, the behavioral sciences. More specifically, for the purpose of this article, exploring the interconnectedness of how we ask questions of animals, and our understandings of their answers, will explain why the capacities of minedogs continue to be contested.

This article is structured around distinct yet historically interrelated approaches to understanding and using minedogs, each taking differing positions on the utility and acceptability of cross-species intersubjectivity, and thus being grounded in highly situated material practices and epistemological assumptions. We begin by reconstructing how dogs were recruited as mine detectors during the Second World War. Long established practices of dog training, such as that espoused by Edwin Hautonvill Richardson, a British pedigree breeder and leading advocate of the use of dogs for military and police purposes, were adapted and redeployed in the making of the minedog. Richardson's approach to working with dogs relied on an assumed, albeit ill-defined, notion of cross-species intersubjectivity. Relatedly, his claim to credibility rested on personal experience and anecdotal knowledge. Consequently, intersubjectivity became a key consideration in early approaches to working with minedogs, while personal experience and anecdotal knowledge formed the basis of the military's early promotion of minedogs. The article then contrasts two contemporaneous postwar scientific investigations of the abilities of minedogs, one British and one American. The former was led by Solly Zuckerman (1904–1993), a medically trained anatomist, physiologist, endocrinologist, and expert in animal behavior. The latter was undertaken by the parapsychologist Joseph B. Rhine (1895–1980). Both men, leaders in their respective fields, visited one another's investigations, shared techniques and research findings, yet arrived at opposed conclusions.

Zuckerman came to believe that dogs were of no practical use for the detection of buried landmines. Rhine, in contrast, resolved that with the right training dogs could be trusted in this role. These different positions will be explained by reconstructing how, in each case, distinct epistemological and ontological assumptions shaped understandings of, and approaches to, scientific knowledge, experimental practice, human-animal relations, and, ultimately, what a minedog should and could be. For Zuckerman, intersubjectivity was a source of variance that threatened to undermine experimental objectivity as well as the dogs’ reliability in the field. Consequently, intersubjectivity had to be excluded from the scientific investigation as well as the practice of working with dogs if they were to become trusted landmine detectors. Pursuing this goal led Zuckerman to police boundaries between human and dog worlds, to repeatedly emphasize differences between human and dog, and insisting both be framed as distinct (and interchangeable) individuals. Rhine, however, approached intersubjectivity as a space to be productively managed as opposed to erased. Rather than policing boundaries between human and dog Rhine sought ways to overcome differences, merging the human handler and dog detector within a “team.” Rhine, therefore, was attentive to the dynamic identities shared by human handler and minedog, recognizing these to be emergent and interdependent. Due to his epistemological commitments to parapsychology, and consequent understanding of ESP, Rhine, unlike Zuckerman, prioritized the study and management of “inside connections.” As a result, Rhine reached quite different conclusions to Zuckerman as to what a dog could do.

By reconstructing these different orientations toward cross-species labor, and explaining how they structured beliefs about how best to work with and understand dogs, this article reveals how orientations toward intersubjectivity, as much as the relations thereby produced, have shaped perceptions of the work that could, and could not, be achieved with dogs. In conclusion, it is argued that perceptions of the animal, other and the relations that follow, have more than instrumental consequence. How we relate, equally, if not more so, has ethical consequence.

## The Making of the Minedog

Despite having played a prominent role in the First World War, on the outbreak of the Second World War both the British and American military had come to view the use of war dogs as antiquated. In contrast, Germany had maintained a national war dog program providing specialized dogs trained to a high standard of performance (U.S. War Department, 1942). In 1940, the British responded to the growing perception that dogs were making important contributions to the German war effort by reestablishing a War Dog Training School (Lloyd, 1948; Richardson, 1950). The British public, partly motivated by difficulties in feeding their animals under rationing, enthusiastically answered a War Office recruitment drive by donating 7,000 dogs for military training (of which a third were deemed suitable for service).^2^ As Edwin Hautenville Richardson, the foremost British expert in dog training and originator of the British First World War Dog program, was judged too old to reenter service, Herbert Summers Lloyd, a champion pedigree breeder, was appointed technical expert and chief trainer in his stead (Richardson, 1910, 1923; Lloyd, 1948). Lloyd, nevertheless, adhered to Richardson's principles of training and working with dogs. By the time the USA joined the war in 1941, war dogs were again an integral part of military life, widely employed in messenger, patrol, and sentry duties. On joining the war in 1941 the U.S. Army, possessing a mere 50 dogs used for pulling sledges in Alaska (Lemish, 2008, p. 33), compiled substantial information on British, French, German, and Japanese use of war dogs, which was used to revive the U.S. training program (U.S. War Department, 1943, p. 7).

Consequently, when Allied Forces first encountered nonmetallic (or low metallic) landmines in North Africa during 1943, dogs were available to face this new threat. Hitherto, landmine construction had necessitated enough metal to trigger the widely sued “polish” metal detector (Croll, 1998). The danger posed by nonmetallic mines led to three hastily innovated responses: tentative prodding, spraying of water to detect disturbed soil, and the use of dogs. Water spraying was only feasible on dry soil without vegetation, while prodding, with typical British understatement, was judged “very dangerous.”^3^ As neither method was entirely attractive, soldiers pressed dogs into service on the basis that they might locate landmines “as they do buried bones” (Zuckerman, 1988, pp. 150–151). Initially, the training of minedogs was conducted *ad hoc* in the field by adapting established principles that had been set out by Richardson for the production of sentry, messenger, and patrol dogs. Somewhat later, the War Dogs Trainings School (Potters Bar) systematically developed a standard regime for minedog training. While little evidence remains of the precise studies that underpinned the new training system, a satirical cartoon by a member of No. 2 Dog Platoon suggests that a wide investigation was undertaken that focused about the olfactory capabilities of dogs (see Figure 2). As we shall see, this illustration is notable for the way it anthropomorphizes the animal actors, particularly pertinent is the cow commenting on the soldier's work, “boring, isn't it son?”

**Figure 1:**
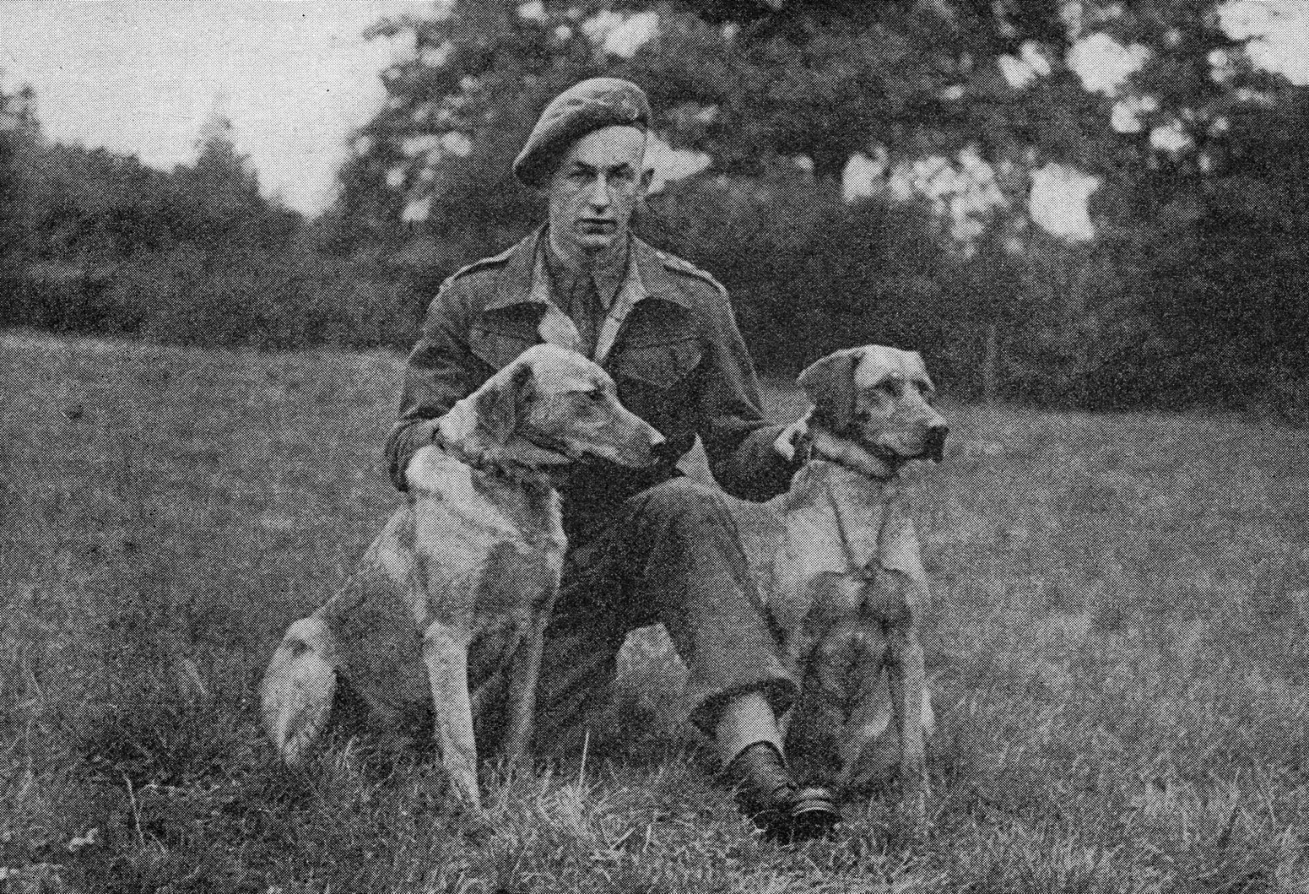
Lt. Peter Norbury, c.1944 with two trained minedogs (Lloyd, 1948).

**Figure 2:**
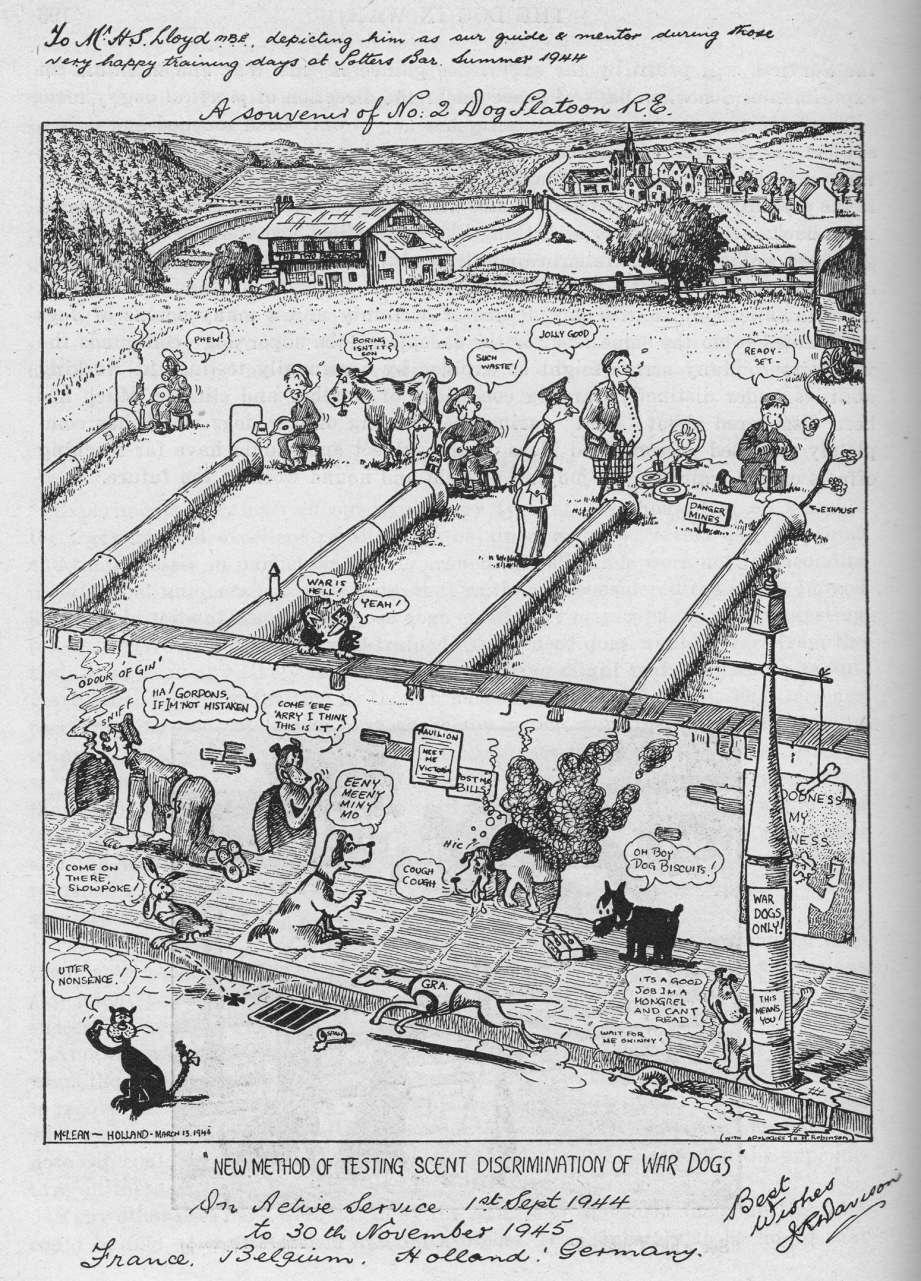
Satirical portrait of activities of Dog Platoon No. 2, summer 1944 (Lloyd, 1948, p. 194).

Military training regimes presumed that dogs orientated their behavior predominantly through their superior olfactory sense. This principle was adapted in the making of minedogs; training was designed to build an association between the odor of meat, the odor of a landmine, and a food reward. The production of a fully trained minedog took on average three months and was structured about four training stages. First, dogs were habituated to wearing a four foot leash and “working harness,” which served as a material means for the handler to communicate to the dog that they were now at work. At this stage, dogs were expected to learn to explore a predefined area where deactivated landmines had been strewn each with a piece of meat (or “chappie”) placed on its top. Dogs were taught that locating and sitting alongside a mine would lead to the “reward” of being allowed to eat the meat. The second stage was similar, except mines were buried with only their very top above ground upon which the meat reward rested. In the third stage, landmines were first rubbed with meat so as to pick up the scent before being fully buried (the reward was carried by the handler and given if the dog sat by a correct burial point). In the final stage, the landmines were buried as they would be in the field with no prior exposure to the scent of meat. By this point the dog had learned to associate the meat reward with the landmine scent, an association that once established was presumed to be retained indefinitely subject to periodic reinforcement. The British approach emphasized reward alone, errors, or so-called “false points,” were investigated by the handler without punishment.^4^ Abstention from any form of punishment, or avoidance conditioning, was derived from the legacy of Richardson's approach to dog training. For Richardson, the maintenance of a positive relationship between human and dog was the guarantor of successful communication and cooperation.^5^ The introduction of a negative association between handler and dog would serve only to prevent the formation of a productive working relationship.

## Working with Mind Dogs in a Shared World

The most important step for facilitating productive cross-species intersubjective working relations was, in Richardson's view, the correct pairing of the subjective characters of handler and dog. Other factors, such as social background of handler or breed of dog, were of much less importance. Indeed, breed was only a consideration in terms of practical and economic needs. In 1944, for example, British army minedog guidance explained the pros and cons of breed as follows: “[s]maller dogs are handicapped in grass and undergrowth, but are useful on roads. Large dogs show no particular advantage … and occupy more space in transport.”^6^ In principle, any breed could be trained for war work, with mongrels being particularly adaptable to service. Richardson, for instance, despite a strong preference for Airedale Terriers, repeatedly insisted any dog with proper character could undertake the work. As guidance on selecting minedogs was notoriously ambiguous, a multiplicity of contested claims as to which breed was best, all based on anecdote and personal experience, emerged. One British report of 1944 claimed that “all breeds can be trained, but the best types are cocker and springer spaniels, and basset hounds.” However, by 1947, “the experience of officers and men in the Dog Platoons” had led to the revised opinion that “for mine detection Labradors and Labrador crosses are likely to be the best.” There was, in fact, good reason for this ambiguity as the majority of military dogs were of uncertain backgrounds. From the perspective of those, such as Lloyd and Richardson, who in civilian life were expert pedigree breeders, the majority of war dogs were “so cross bred as to rarely show the characteristics of one breed predominantly.”^7^

Consequently, discourses of breed, hereditary, or genetics, though appearing in informal discussions, could not reliably serve as conceptual or material reference points in war dog training. Instead, dog identity was built about individual “character.” In selecting a dog, instructors were advised to:

look for an intelligent expression and one broad across the nostrils which, always slightly quivering, denotes the dog is using his scenting powers unconsciously and his sense of smell is usually highly developed. Dogs with black eyes are frequently sulky and erratic in their work, whilst dogs with light eyes, are generally wilful and inclined to use their eyes in preference to their nose. A dog with a hazel-coloured eye, usually has the firmer character, and for this reason should be selected.^8^

In this way, physical markers were established for desired characteristics that translated to presumed performance. Unlike breed, this practice had the benefit of not affronting the values of pedigree breeding by ascribing purity to a mongrel. Nevertheless, it too rested on experience and anecdotal knowledge, and, unlike pedigree, had no paperwork to justify its claims. The most important characteristic in the minedog was known as “willingness” or “keenness,” terms that captured the otherwise ambiguous capacity of a dog to respond to the requests of their handler, and only their handler. A dog “ranked high in willingness if he persistently responds to his master's request,” yet would “be penalized in his rating on this trait if he readily shifts his ‘willingness’ to another man” (U.S. War Department, 1943, pp. 14–15). This illustrates the extent to which military practices prioritized the establishing and managing of an intersubjective bond between a specific handler and a specific dog. Military guidance, therefore, represented the dog less as an interchangeable technology and more as an individual forming an integral part of a “man-dog team.”^9^

A successful minedog, then, relied on the maintenance of a positive intersubjective bond between handler and dog, which, in turn, necessitated matching the right individuals to form the team. In practice, training and working with dogs was predominantly a question of productively managing relationships, at the heart of which was an always ambiguous notion of intersubjectivity. Working with animals, Richardson believed, required “a decidedly special gift in the instructor” (Richardson, 1920, p. 64). The precise nature of this “gift,” though always vague, was most often described as “a natural love of animals” (Richardson, 1920, p. 65). Accordingly, military guidance emphasized the importance of choosing men who were “fond of dogs” as this indicated that the man could productively relate toward the dog.^10^ Similarly, for dogs, the aforementioned biological markers provided a guide to choosing animals with suitable temperaments. Once established, the working partnership was considered unique and thus sacrosanct. With the exception of the handler, others were forbidden from any interaction with the minedog for fear of disrupting the intersubjective understanding of the team. The belief that minedogs would “be completely ruined if all and sundry try to make friends with them” led to the “One Man, One Dog” rule. This categorically stated that a dog be paired to one handler and expected to work only with this “master.”^11^ Indeed, the intersubjective relationship was so important that any infringement of this rule, even by a soldier merely petting or greeting a dog, was “treated as a serious military offence.”^12^ Even handlers were expected to carefully manage their emotions at all times; their success and their lives depended “primarily on quiet, sympathetic handling” as “nervousness or lack of concentration on the part of the handler has an immediate reaction on the dog.”^13^ As their mood could easily unsettle the minedog, handlers were instructed to “never work a dog when feeling out of sorts with yourself.”^14^ Collectively, these practices contrast sharply with the conventional emotional landscape of military life, where discipline and regimentation was the norm.

While working with dogs relied upon overcoming difference by bonding handler and dog so as to facilitate intersubjective understanding between the two, other aspects of minedog knowledge operated to instantiate otherness. For instance, humans and dogs were understood to inhabit different sensory worlds. As one U.S. Army manual explained:

The dog's world differs from the human in specific ways. His world is predominantly one of odours. His nose tells him countless things about the environment that entirely escapes humans. He is more sensitive to sounds. His vision is considerably inferior to human vision, and for this reason he depends less upon it (U.S. War Department, 1943, p. 9).

This reflected wider cultural impressions of the time that presumed dog worldliness to be divided from and distinct to human experience and identity. Freud's seminal account of animal-human difference, for example, asserted that primal man, like the dog, existed within an olfactory world. According to Freud, the “threshold of human civilization” occurred only when man repressed the dominance of olfactory stimuli in favor of the visual world (Freud, 1930, pp. 66–67). Following this logic, the combination of human and dog sensory worlds would produce a team that was more than the sum of its parts. Species difference and identity, thereby, provided a conceptual justification for the creation of the minedog. As a team, man and dog could enter into a shared sensory world that would provide superior access to reality.

Working with dogs, therefore, was conceptually, practically, and experientially different to existing practices of locating mines using a mechanical mine detector. Once trained, the operative and Polish metal detectors were considered interchangeable. In contrast, British and American wartime guidelines repeatedly emphasized how important it was to remember that “[m]ine detection by dogs is a team-work job.”^15^ It required a new and unique recognition of interdependence; mine clearance became a lived and shared experience. To the uninitiated, minedogs were mysterious and unpredictable, not least due to their known tendency to “stop working without appearing to.”^16^ Only the experienced handler, sensitive to the subtle working habits of their individual dog, was judged capable of interpreting this behavior and thus recognizing when, and when not, to trust the minedog. Success demanded the careful management of relationships so as to facilitate communication across the sensory worlds inhabited by human and dog. Lives depended on the creation and maintenance of a shared fluency in a unique intersubjective language. A language, moreover, that was necessarily ambiguous to the outsider. Trust, in sum, was not placed in the dog alone. Rather, it lay with the dog-handler team.

## Working with Minedogs in the Field

Minedog Platoons were institutionalized across the Allied armies by 1944. However, rather than improving with experience, their increased use revealed many difficulties and inconsistencies:

being living animals, and having living handlers, dogs are temperamental and do not always work to the same standard: and if not carefully treated, very easily become fatigued, and even may refuse to work completely. Hence a dog which may have been graded Good at one school, may not perform nearly as well when assigned to a mine detection platoon.^17^

Nevertheless, while accounts of dogs refusing to work in the field were rife, faith in the minedog remained strong. No field report refuted the ability of dogs to detect landmines. Rather, they described, in diverse ways, the challenge of encouraging dogs to work in a constantly changing environment. All agreed that a dog might work well at one time and poorly, or not at all, at another. There were many explanations for inconsistent performance, but all shared a tendency to identify the problem as located in the interactions of handler, dog, and environment. Thus, it was never that the dog was unable to detect landmines. Rather, difficulties were presented as a problem of inadequately managing dynamic relations, or, relatedly, as difficulties in communicating between handler and dog. Accordingly, the key minedog characteristic, “keenness” for the job, was understood less as a biological trait but something that was learnt and thus had to be maintained. Mine detecting, in the words of one Dog Platoon leader, was:

an entirely new departure, and … by far the hardest task yet asked in training dogs for war purposes. The keen-ness for the job has to be taught as it is not a hereditary trait like gundogs, or sheepdogs possess.^18^

Field experience increasingly revealed that sustaining keenness across varying, often arduous environments, events, and psychosocial experiences, was a significant challenge.

Mine clearance platoons traveled widely, across shattered transport infrastructures, often during or close to on-going warfare. Confinement, coupled with vibration and noise during travel, affected dogs in distinct and unpredictable ways. Most commonly, dogs were described as becoming “listlessness,” or developing a “sickness” that could only be overcome by time, patience, and exercise after the journeys end.^19^ Similarly, dogs responded in an assortment of ways to seasonal variations. Sudden changes in climate weather, and particularly temperature, unsettled some dogs. Furthermore, the war presented numerous additional environmental variables to contend with, from unexpected sounds to a vast range of shrapnel and battle refuse. Of course, these obstacles were often only known to the handler, thus were not subject to systematic categorization. Moreover, there was no certainty that a dog would react to the same change in the same way. Nonetheless, a common frame for explaining loss of keenness in action eventually emerged. Differences between training and field experience were identified leading to new training techniques conceptualized as “battle inoculation” designed to better model the realities of mine detection. Training was adapted so that dogs were exposed to sounds, including gun fire, shouting, explosions, machinery, and engine noise. The hitherto clean training fields were altered so as to be littered with shrapnel, shells, cartridge cases, and other battlefield waste. Nevertheless, the variability of dogs always allowed for disagreement. Some handlers, for instance, complained that introducing noises and shrapnel confused the dog, while others claimed that asking dogs to distinguish between shrapnel and buried landmines was straining their sensory capacities (Lloyd, 1948, p. 188).

Several problems, however, only emerged in the field and could only be corrected there. Experience quickly revealed the need to immediately isolate a bitch coming into season, for example, as otherwise they would distract the entire platoon. Local variations in landmine design and explosive type also presented difficulties. As with other forms of field underperformance, the common response was training reenforcement, using purpose laid minefields designed to model local environments.

This acclimatized dogs to new environments and was also thought to boost declining performance rates in experienced dogs. However, some handlers argued that this was counterproductive as it could encourage dogs to associate failure in the field with a move back to training where reward was more easily gained. Yet others believed it necessary to expose dogs to the comparatively easy challenge of training, as dogs lost interest in their work if they did not regularly experience the reward of finding a mine. This again reveals the remarkable scope for flexibility in interpreting the best way of accommodating the needs of dogs. Consequently, over the course of the Second World War, the principles governing work with minedogs became increasingly diverse, with practices proliferating in response to local challenges. From the perspective of the War Office, the fast multiplying reports of good practice received from different dog platoons appeared contradictory and inadequately justified. Mine clearance was a high-risk activity, as a 1944 British memoranda explained:

[o]pportunity only knocks once, and a mine missed by the dog and handler simply spells disaster for both. Play for safety all the time.^20^

The question of just how best to play safe when working with minedogs grew increasingly urgent as proliferating and often conflicting reports of best practice were received from the field.

The need to systemize knowledge so as to produce official guidance was of critical importance to the War Office at two levels. First, the high-risk nature of mine clearance required reliable standards of practice. Second, producing minedogs entailed a considerable investment of time, money, and manpower, as did the logistical commitments to their transport, husbandry, and long-term keeping. As the challenge of mine clearance continued after the close of the Second World War, Major R. Cawthorne, a Royal Engineers Veterinary Officer, was tasked with collating what was presently known of minedogs and establishing, scientifically, the best means of their training and use. In 1946, Cawthorne interviewed a number of officers from the British Army Dog Training School, as well as those with field experience, and studied existing reports, before concluding that an extensive experimental program was required to subject conflicting claims to scientific scrutiny.^21^ The result was intended to provide a definitive and systematic understanding of minedogs. Instead, his investigation revealed only the difficulty of establishing any universal rules on how best to work with dogs, or even assess their capabilities.

Cawthorne began with an attempt to scientifically test the presumption that dogs detected landmines using their olfactory senses. Six trained minedogs were chosen; four had their olfactory nerves surgically severed so as to abolish their sense of smell. The other two acted as controls, subjected to the surgical experience without having their nerves cut. If the olfactory hypothesis was correct, the dogs that had been surgically deprived of their senses were expected to be unable to detect landmines. Yet, when tested the surgically altered animals suffered only a minor performance decline. Had surgical deprivation failed? Cawthorne tested this possibility by exposing the animals to odorous substances known to cause extreme distress. Their nonreaction seemingly confirmed their state of olfactory deprivation. To be sure, in an echo of Freud, Cawthorne introduced the same dogs to a bitch in season, believing this scent to be an extreme test of olfactory deprivation, only to find the dogs reacted normally. Cawthorne lacked a way to reliably translate a dog's olfactory experience so as to be humanly comprehensible in a form that allowed for a consensus of interpretation. However, opening up the dogs’ sensory world to experimental analysis proved challenging. One approach, taken on the advice of the physiologist Edgar Douglas Adrian, was to trial the electroencephalograph (a then novel device for recording changes in the electrical activity of the brain) as a means to make visible the brain's response to olfaction. Even with this technology, Cawthorne could not meaningfully reconcile brain response and the varied performance of the experimental minedogs so as to establish the olfactory hypothesis. In 1947, after a year of frustration, Cawthorne suffered a nervous breakdown bringing his work to a premature end.^22^ As a result of this failure, the British military adopted a more cautious position, describing minedogs as “unreliable” due to their “temperamental” nature.^23^ Nevertheless, mine clearance remained an urgent postwar priority.

## Solly Zuckerman: Working with Minedogs in a Divided World

Given the evident complexity of the problem, the War Office concluded that a “trained scientist” should be recruited to establish “the facts” about minedogs once and for all.^24^ Solly Zuckerman was the scientist selected. During the war, Zuckerman had become a prominent figure in government and military circles by driving the mobilization of British science to serve the war effort.^25^ He was a medically trained anatomist, physiologist, and endocrinologist, who had first established his scientific name in the field of animal behavior (Zuckerman, 1932). In 1936, Zuckerman had been a founding member of the Institute (now Association) for the Study of Animal Behavior subsequently maintaining a keen interest in the development this field (Durant, 1986). Moreover, as an architect of “operational research,” Zuckerman had garnered significant military credibility, most famously an analysis of the impact of air raids on civilians which, controversially, concluded that intensive bombing was unlikely to be detrimental to morale (Burney, 2012). He had also investigated the effects of blast, producing innovative studies of the then little understood physics of explosion and its effects on the body (e.g., concussion and the tendency for wounds caused by high-velocity fragments to be disproportionate to fragment size). This work, which utilized human bodies (those injured in air raids) and animal experiments (rhesus monkeys), contributed the development of preventative measures against blast. Indeed, as he had directly studied the effects of landmine blast during the war, few understood better the devastating consequences of triggering an undetected mine.^26^ Zuckerman's rare ability to seamlessly traverse the work cultures of science, military, and state bureaucracies was well known, as was his capacity to produce answers to complex problems. In short, in the view of the War Office, he was ideal for the job.

Zuckerman had recently joined the University of Birmingham as Professor of Anatomy. From his perspective, investigating minedogs provided an opportunity to establish what he called “Experimental Animal Psychology” at the University.^27^ Zuckerman had in mind from the start a small team, which he would lead (mobilizing a talent for scientific delegation mastered during the war). Finding suitable assistants, however, was challenging as at this time the pool of qualified graduates in animal behavior was very small. Moreover, the research was unattractive as publication could not be guaranteed due to the work being classified under the Official Secrets Act.^28^ What delayed the project's start more than anything else was Zuckerman's steadfast insistence on the right candidate. In particular, he refused outright to consider any person tainted with the field of ethology, a then fast emerging new approach to the study of animal behavior that Zuckerman disliked due to a perceived absence of experimentation. Though the contract was awarded in 1948, work only began in 1951 after Zuckerman abandoned his search in favor of compelling Eric H. Ashton, a newly graduated Birmingham PhD, to take on the field work. As a Birmingham student, Ashton, Zuckerman was sure, could be trusted not to introduce ethological approaches to the proposed study. John T. Eayrs, a recently appointed lecturer to Zuckerman's department, discovered soon after appointment that one of his roles was to maintain communication between Ashton and Zuckerman.

In this way Zuckerman maintained tight control over the design and activity of the experimental investigation, directing each stage while leaving the day-to-day work to assistants. This was, in part, a pragmatic necessity. Zuckerman lacked the time to be out in the field training dogs. He was busy institutionalizing himself at Birmingham and across the higher echelons of scientific society, while simultaneously building a governmental influence in Whitehall that would see him appointed as the first Chief Scientific Advisor to the government in 1964. The experimental field work was scheduled to be conducted in remote locations, first at the Royal Army Veterinary Core's War Dog School (Melton Mowbray) and later at the Signals Research and Development Establishment (Christchurch). The isolation involved, from a personal and academic perspective, was deeply unattractive even for Ashton who insisted on being transferred back to the comfort of a University laboratory in 1954 (where he studied olfactory thresholds of dogs). Subsequently, David Gillman Moulton, a newly qualified zoologist taught by S. A. Barnett at the University of Glasgow, was appointed to conduct the fieldwork.^29^ Though largely a pragmatic arrangement, this organizational structure instantiated a principle that was to guide Zuckerman's entire experimental investigation, namely the distancing of human from minedog.

As a condition of taking on the work Zuckerman had insisted the War Office alter its “premature” position that “dogs can be trained to detect mines.”^30^ When working with animals, Zuckerman explained, “the possibilities of self-deception are very much greater than with non-living systems.”^31^ Nothing should be assumed that has not been experimentally proven. Whether dogs could, or could not, detect landmines, was an experimental question and not a given to be explained. According to Zuckerman, allowing unfounded assumptions to shape experimental design led only to error. Once anecdotal knowledge was ruled out, what hard evidence was there that dogs could detect landmines? Scientifically, one could never explain a “how” until the existence of the phenomenon itself was established. For Zuckerman, to allow unsubstantiated anecdotal knowledge to shape experimental design was contrary to scientific method—none more so than for animal behavioral research. Cawthorne's mistake, ascribed by Zuckerman to his lack of “experience of the snares and pitfalls of experimental work on behaviour,” had been to allow his initial experimental premise to be shaped by anecdotal reports from the field.^32^ In contrast, Zuckerman distanced his investigation from the anecdotal understandings of minedogs. Simultaneously, he excluded from his experimental design all influences he considered to be inexplicable to science. Foremost among these was the idea of the dog as a knowing subject. The ideal minedog would perform with mechanical consistency regardless of changing local circumstance. Minedogs were conceptualized mechanically, operating literally as automatons. Zuckerman insisted that the minedog be imagined as moving in response to the material stimuli of the mine alone. Behavior was to be understood, explained, and manipulated without reference to subjectivity. From this perspective, dogs could only be trusted to detect landmines if they could do so consistently, with experimentally demonstrated reliability. More than anything else, it was this radical conceptual commitment that led Zuckerman to conclude that minedogs were of no practical value.

Zuckerman's vision of the ideal minedog mirrored, conceptually and materially, the needs of the War Office. For a mixture of economic and practical considerations relating to the danger of mine clearance, the War Office demanded a minedog that would operate as machine-like as possible, operating in the same way regardless of changing user, local environment, or seasons. Briefly, therefore, Zuckerman considered developing a means of “translating the performance of the dog to some hypothetical instrument.”^33^ Such a feat would have required building a mechanical device capable of detecting yet to be isolated material property common to all landmines that the dog was responding to. Zuckerman quickly realized that this was beyond the ability of the science of his day as “the sensitivity of our animals is very much greater than that of any known instrument which can detect vapours.”^34^ Consequently, the next best thing was to transform the minedog in to as close an approximation of an automaton as possible. This agenda closely resonated with Zuckerman's vision of experimental psychology more generally. Experimental organisms were expected to respond to the same scenario in the same way so as to facilitate experimental replication. Minedogs, whether functioning as a mine detector (in the field) or an experimental organism (in Zuckerman's investigation), were framed in the same way. Both roles placed value on a dog whose behavioral repertoire was restricted so that responses to specified stimuli remained consistent. Ends and means mutually construed the ideal minedog as an automaton, reacting to a single stimulus, the landmine, without reference to the wider physical environmental, social relations, or subjective states.

While Zuckerman's approach reflected wider trends in the behavioral sciences of the time, he was particularly radical in his insistence that behavior be explained only through reference to biochemical, physiological, or other material components. At no stage were potentially subjective factors, up to and including the social environment, considered for use as a productive tool for manipulating the dog. Instead, Zuckerman's experimental designs were framed entirely about physical components. Subjective responses and affective relations were considered only as sources of variance to be controlled and/or erased so as to guarantee experimental validity (cf. Dror, 1999). These epistemological commitments led Zuckerman to reject all conceptual or explanatory frames that invoked subjecthood. This included the intersubjective bond between handler and dog that had hitherto been central to working successfully with minedogs. In Zuckerman's view, intersubjectivity threatened experimental objectivity (i.e., replicability) and the practical utility of minedogs. Reliable tools did not work for one person over another, or in one climate or time of day and not another. In mine detection, where lives were routinely at stake, this was never more important. Behavioral consistency had to be a key definition of reliability; trust could not rely on insubstantial notions such as subjective relations.

In order to remove intersubjectivity handler-dog relationships were to be standardized. Zuckerman set two criteria for successful standardization. First, handlers and dogs would be interchangeable. Second, dog performance would be predictable and consistent. This approach conceptualized minedogs as automatons, operating within a hermetically sealed world in response to physical stimuli alone. Controlling the physical world therefore became the tool for manufacturing a minedog. This ideal was instantiated through the building of “model environments” that enabled the systematic isolation, identification, and manipulation of dogs’ behavioral responses to specific stimuli. In practice, highly controlled model environments were intended to train dogs by gradually narrowing their sensory world until it consisted solely of the presence or absence of landmines. The goal was an automatous dog, operating within a model environment, responding only to the material stimuli of a landmine. Within this schema, there was no productive role for the dog as a mindful subject, as author of its own actions. By extension, the intersubjective relationship, hitherto central to military practices, was to be actively erased as a condition of experimental validity and minedog utility.

In recent years, *model organisms* have become of increasing interest to science studies scholars. In contrast, *model environments* remain relatively understudied. In part, this is an effect of the scholarly focus on biomedical experimental systems, where laboratory practice is structured about the manipulation of organisms with the aim of “modelling” a specific pathology or disease. Historically these practices mobilized inbreeding as one of many techniques to “standardize” organisms (e.g., Clause, 1993; Kohler, 1994; Rader, 2004). More recently, catalyzed by advances in genetic science, a comparable agenda underpins the biomedical production of so-called “model organisms” (cf. Ankeny & Leonelli, 2011). While not averse to manipulating organisms, the behavioral sciences tend to take a different approach, manipulating both organism and environment in the production of experimental systems. The Pavlovian behaviorist, H. S. Liddell, for instance, developed what he termed “skeletal psychological environments” as a key tool for investigating animal behavior (Liddell, 1947, p. 579). Model environments, which accentuate the design, construction, and standardization of environments *as tools of experimentation*, have yet to be analyzed on a par with that of model organisms (cf. Ramsden, 2011). Nevertheless, the environment has long been a critical consideration even for organism-focused experimental systems (Kirk, 2009). This is particularly important for the behavioral sciences, where appeals to a range of factors across the physical and social environment demand a much more complex and dynamic process to establishing experimental validity. Here, the experimental organism becomes one component within a much broader experimental system encompassing the entire physical and social environment which, in the most reflective of cases, could include the researcher (cf. Gantt et al., 1966).

**Figure 3:**
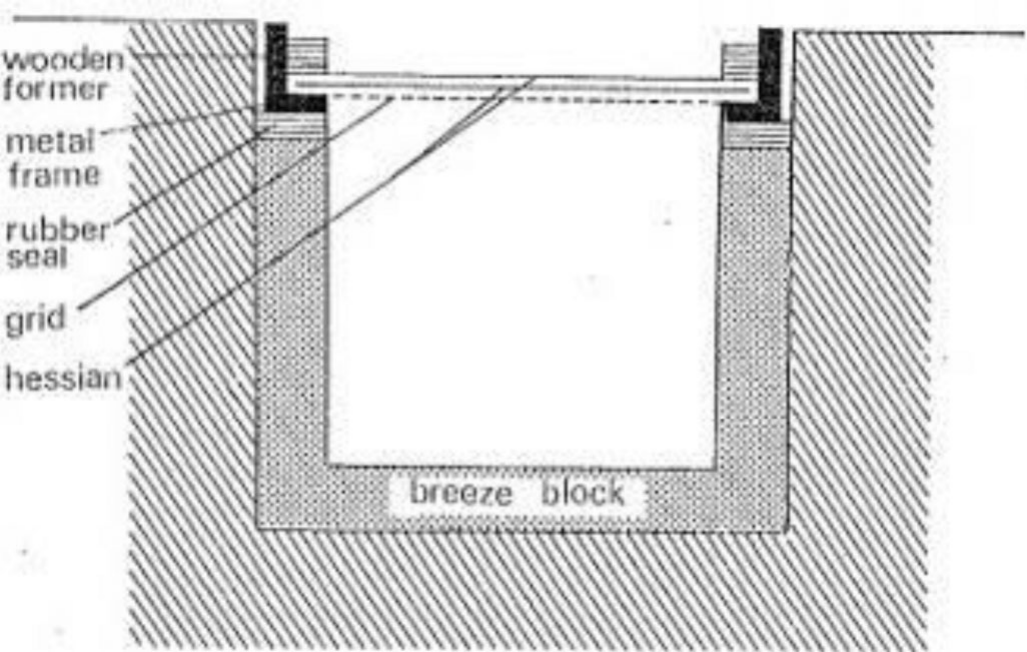
Breezeblock well (Ashton & Eayrs, 1970, p. 253).

Zuckerman, however, refused to consider interpersonal relations between researchers (or handlers) and animals (or minedogs) as acceptable experimental tools. Instead, subjective relationships were cast as uncontrolled and uncontrollable variables that had to be erased through the model environment. Zuckerman's investigation operated by presenting minedogs with “stereotyped situations” (model environments), designed to simulate aspects of minefields.^35^ The basic model consisted of a run of four wells, each lined with breezeblock and sealed by metal grid covered with hessian cloth (Figure 3). Eliminating the act of burial simplified and standardized the environment by removing stimuli linked to disturbed soil, with the aim of revealing whether or not dogs possessed the ability to respond to unseen stimuli alone. Dogs demonstrated a remarkable capacity to detect a variety of objects concealed in the wells, including wooden and metal cubes, glass dishes, and landmines. This basic test established that dogs could, in principle, detect unseen objects. Moreover, as success rates diminished with the size of the hidden object, Zuckerman inferred that the mode of detection was material, most likely a chemical detected by the olfactory senses. From this point, the challenge of experimentally substantiating this olfactory hypothesis dominated his investigation (yet was never satisfactorily achieved).

**Figure 4:**
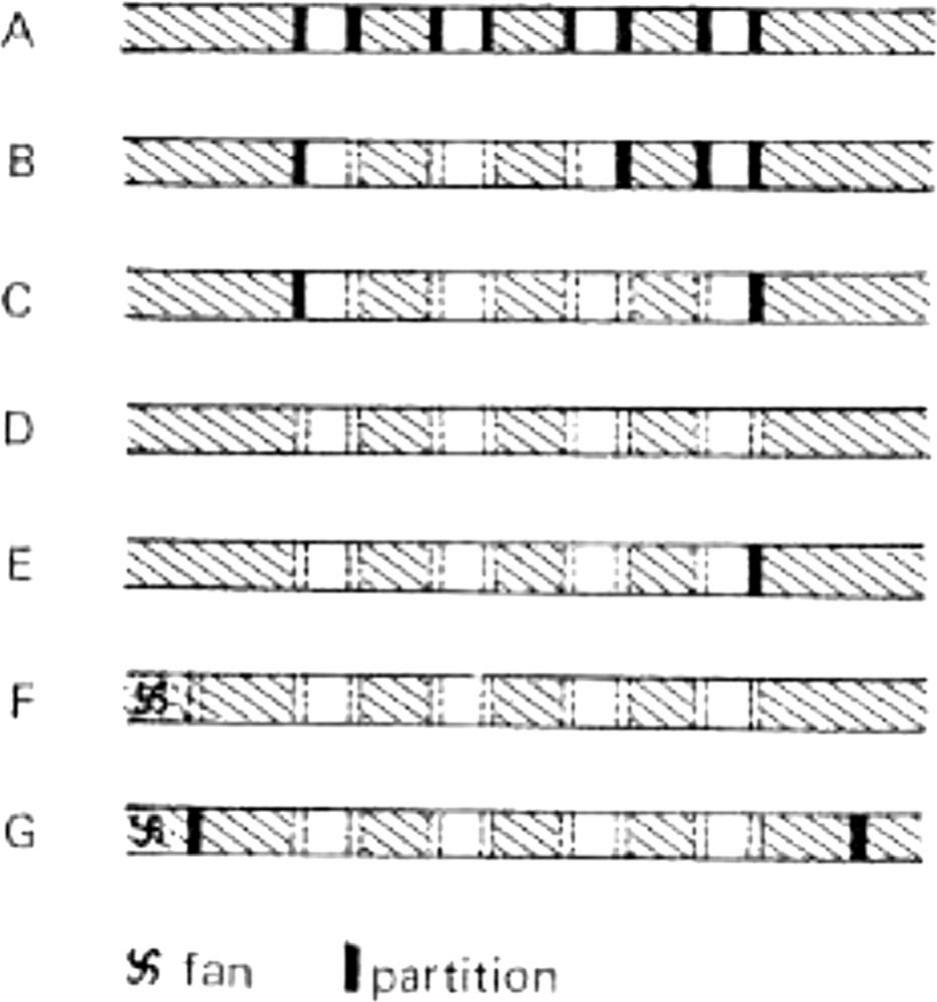
Various tunnel wells setups; (A) four independent wells; (B) and (C) removal of partitions to test effects of increased air space; (D) and (E) further increase of air space by means of open endedness; (F) fan incorporated to control air flow; (G) fan operational but partitioned to prevent air flow in order to test effect of fan noise (Ashton & Eayrs, 1970, p. 256).

An early approach to testing the olfactory hypothesis involved contaminating wells with strong masking odors (e.g. aniseed) with the expectation success rates would decline due to the stimuli from the landmine being obscured. Contrary to expectations, no odor produced a consistent effect. Some dogs were hindered by one odor and not another, while others could be entirely unaffected. Moreover, individual dogs responded to the same chemical differently at different times. A second experiment, where dogs were challenged to detect breezeblock lumps that were identical to the blocks used in the well construction, produced yet more confounding results. If the stimuli was a chemical detected by the olfactory senses the task should have been impossible. Yet all dogs demonstrated a strong capacity to detect breezeblock within breezeblock wells. How could dogs discriminate the absence of presence of a scent if the hidden object was the same material as the walls of the wells? Not only had this experiment failed to provide evidence to corroborate the olfactory hypothesis, it appeared to undermine it. Yet, Zuckerman was reluctant to abandon the olfactory hypothesis, primarily as it promised a material explanation for the dogs’ ability. Consequently, counterarguments were proposed to invalidate the breezeblock experiment. The model environment was now reinterpreted, rather than having simplified the environment it was suggested that the artificial situation had introduced new as yet unknown stimuli. Initially, the breezeblock results were explained away on the presumption that the dogs had learned to discriminate full from empty wells by listening for sound resonance, a factor that had been inadvertently introduced by the model environment. However, when Moulton was appointed to replace the exasperated and exhausted Ashton in 1954, he conducted careful sound recordings that showed that there was little if any change in the sound frequency of full and empty wells. By this point, however, the olfactory hypothesis had been satisfactorily established using an alternative model environment. Here, rather than being individually dug, wells were constructed from movable partitions located within a roofed tunnel (Figure 4). This design allowed for the manipulation of air space within each well by the removal of partitions. Airflow could be controlled using electric fans placed at the end of each tunnel. Ashton had run a series of tests that had shown performance to improve when airflow was concentrated toward a well and decline when airflow was directed away from the well. Zuckerman interpreted this to mean that the dogs were responding to a material, likely chemical, stimuli, emanating from objects and traveling by air. The breezeblock results were quietly forgotten.

Of course, the fact that dogs could detect hidden objects did not mean that their ability had practical utility in the field. The act of burial might well prevent molecular diffusion. Assessing practical utility required new model environments that better represented the reality of mine detection in the field. In the first new environment, landmines were laid according to standard military practice, as were so-called “dummy” sites where soil was disturbed but no object buried. A second model was created by entirely stripping topsoil from a field, laying mines, then replacing and smoothing the soil so as to leave no visible indication of burial.^36^ Minedogs were found to perform with less success in the first new environment than with the wells. Deprived of visible cues in the second model, dogs proved of no practical value whatsoever. Further experiments, for instance testing dogs on fields where mines had been laid for differing length of time, suggested that dogs were predominantly reacting to recently disturbed soil as opposed to the landmine alone.

In 1956, Zuckerman concluded that dogs were of no practical use for mine detection. Once buried, soil increasingly smothered a landmine's olfactory stimuli over time. Apparent success with the well and tunnel environments, alongside anomalous successes with individual dogs on the model minefields, was explained away by the presumption of visible indicators. Indeed, visible “choice points” were said to be an essential stimuli to which the dog responded by indicating the presence or absence of a landmine (Aston & Eayrs, 1970). Without the visible indicator, which may or may not be visible to handlers, dogs could not respond as though they were detecting a mine. This explanation allowed Zuckerman to dismiss the substantial number of anecdotal reports of success with minedogs in the field. During the war no effort had been made to statistically analyze performance. Had this been undertaken, and false points ruled out, Zuckerman surmised that the detection rate of minedogs would have amounted to little more than that expected from chance. In 1970, when declassified and published in full, the distance between initial success and subsequent failure was rhetorically widened:

the level of the dog's performances shown by these experiments provides little support for some of the earlier claims that the dog could be of practical value as a detector of concealed or buried objects (Ashton & Eayrs, 1970, p. 260).

This was the basis of Zuckerman's claim that “dogs could never be the mine hunter's best friend.” Nevertheless, at the 1969 Ciba Symposium on Taste and Smell, the attentive audience seized upon the apparent divide between findings and conclusions. One discussant queried how, if the dogs could detect breezeblock lumps within breezeblock wells, one could conclude they were operating by olfactory senses. Having failed to evade the question, Ashton and Eayrs coyly admitted that “we were never able to accumulate enough information to give a real answer” (Ashton & Eayrs, 1970, p. 263). The problem, however, was never lack of information. On the contrary, the minedog investigation was hindered by an abundance of contradictory information.

## Relating to the Dog as Automaton

By making the goal of the minedog as an automaton dependent on the erasure the dog as a subject and author of action, Zuckerman placed the former in direct conflict with the latter.^37^ Yet, the degree of behavioral control Zuckerman sought proved impossible to realize. His investigation was continuously hindered by inexplicable behavioral inconsistencies that resisted the explanatory framework of physical environment. Minedogs would not perform as automatons, indeed they proved consistently inconsistent, working successfully in one instance and not at all at another. Nevertheless, Zuckerman held firm to the conviction that behavior be managed without reference to the subjective or affective world of the dog. As a consequence of this approach, his experimental trajectory spiraled into an apparently endless cycle of identifying and closing down hidden environmental stimuli within the model environments, where each refinement seemingly introduced yet more complexities demanding further explanation. Moulton, for instance, introduced rigorous decontamination procedures to prevent experimental objects, as well as the new model environments, from becoming contaminated by odors. Years were spent systematically ruling out the possibility of interference from nonolfactory stimuli; utilizing various laborious techniques such as slow-motion film, sound recordings, and blindfolding. The search for physical explanations for behavioral inconsistency led to all manner of environmental variables being considered, isolated, and investigated, including air temperature, humidity, barometric pressure, brightness of sky, reflected brightness on the ground, moisture, temperature, and pulse rate. Frustrated, Moulton even studied whether varying rectal temperature of dogs might discourage sitting. Rather than simplifying the model environment, each investigation of a new factor introduced new complexities, opening up new questions by revealing further behavioral inconsistencies. Despite an exhaustive survey, no quantifiable environmental factor directly correlated with the “marked day-to-day variation in the performance of the dogs.”^38^ How, then, had Zuckerman's investigation arrived at a conclusion?

Surprisingly, a common process of fashioning experimental closure was to invalidate inconsistent results by introducing the dog as a subject. In unpublished reports, circulated between Moulton, Ashton, Eayrs, and Zuckerman, variance in the performance of individual dogs, and of the same individual over time, was explained through ambiguous notions such as “temperament,” which in turn were loosely mapped on to breed:

Differences in ability seem to be correlated with differences in temperament. A placid dog is easier to train and handle and is more reliable. For this reason Labradors and their crosses are preferred, the cross-bred proving more amenable than the pedigree. “Temperamental” dogs like Alsatians, terriers and spaniels are distractible and difficult to train.^39^

Characteristics such as “temperament” were frequently enclosed by quotes, operating to distance the anthropomorphic language from literal meaning. In practice, temperament served a similar function as eye color had in earlier military approaches. It provided a flexible language, loosely tied to biological markers, by which dogs could be distinguished and contradictory behavior explained. Yet, conceptually, reference to temperament operated more as a gesture *toward* an explanation than as *an explanation itself*. Nevertheless, it was a powerful rhetorical tool for bringing about experimental closure, as it ambiguously ascribed and refused dog subjecthood on two levels. First, though it was a common explanatory gambit in the informal language used within internal communications, letters, and reports, reference to temperament was carefully erased from formal reports and publications intended for an external readership. Second, temperament was always framed as a negative factor that was responsible for, and thus capable of explaining away, inconstant behavior.

Invoking subjectivity, therefore, in no way contradicted the aim of erasing the dog as subject in the training of minedogs. On the contrary, the language of temperament framed dog subjecthood as an unwanted intrusion into the experimental scenario, providing a logically consistent narrative to reconcile experimental expectation with observed behavior. This is evident in a series of experiments, ran between 1953 and 1954, designed to substantiate the olfactory hypothesis by demonstrating that the deeper a mine was buried the harder it would be to detect (depth was assumed to better smother the olfactory chemicals). Three minedogs were tested, but only one, Duke, a black Labrador retriever, met with expectation. Frank, also a black Labrador retriever, performed consistently regardless of depth. The third, an Alsatian named Slam, improved with depth. In a rare instance of consistency, all three dogs performed with the same pattern of results time and again. An exhaustive search for distorting physical stimuli revealed nothing. Ultimately, Frank and Slam's anomalous performances were ambiguously disregarded as “more apparent than real.” Anthropomorphic narratives were introduced to recast both dogs as “being nonchalant in their attitude.” In contrast, Duke was described as “reliable” and “attentive,” keeping his “nose to the ground.” All three dogs were simultaneously imbued with biography. Duke was distinguished from Frank and Slam as he was “more experienced,” a rare acknowledgement of the potential importance of a dog's past.^40^ In this way, the dog as subject, complete with biographic history, operated to reconcile observed behavior with experimental expectation. Frank and Slam's performances were disregarded as anomalous whereas Duke, perceived to have been working hard and interested in the task at hand, was judged “normal” thereby confirming the olfactory hypothesis.

Generally, subjectivity was only invoked as a negative factor associated with behavioral inconsistency. While not ideal, this usage was deemed compatible with Zuckerman's insistence that subjectivity had no place within the behavioral sciences. In *The Social Lives of Monkeys and Apes* (1932), the study that had established his scientific name, Zuckerman had forcefully argued that speculation on animal subjectivity was unscientific, leading away from verifiable experimental knowledge toward anecdote and anthropomorphic supposition. He mercilessly critiqued earlier studies of behavior that used animal subjectivity as an explanatory device. That William Lauder Lindsay could claim animals’ possessed “the higher mental faculties as they occur in man” on no firmer basis than the word of “children, school-girls, young ladies, farmers’ wives, and other ladies” made Zuckerman indignant (Zuckerman, 1932, p. 10). Science had to be verifiable and thus experimental. Anecdote was utterly unverifiable, thus should not be confused with science. By extension, anthropomorphism, as the prominent discourse capable of sustaining animal subjectivity in language, was also deemed unscientific. In addition to unjustifiably blurring the categorical distinction between human and animal, anthropomorphism introduced a degree of subjectivity to the encounter between observer and observed. Here, Zuckerman followed his friend, Bertrand Russell, who believed anthropomorphic interpretation was why “all animals that have been carefully observed have behaved so as to confirm the philosophy in which the observer believed before his observations began.”^41^ For Zuckerman, anecdote and anthropomorphism had no role in science. Such approaches were little more than looking at animals as if they were mirrors.

Accordingly, while recognizing evolutionary affinities in material biology, Zuckerman drew a strong distinction between the psychosocial experiences of human and nonhuman animals. Human psychosocial life, Zuckerman argued, was unique in that it is shaped by cultural history. Human psychosocial behavior was exceptional, and infinitely more complex, because the “effective stimuli underlying human behaviour are largely products of the lives of pre-existing people.” In contrast, the “effective stimuli involved in the behaviour of animals are mainly inherent in immediate physical events” (Zuckerman, 1932, p. 17). Zuckerman therefore framed the study of animal behavior about the principles of anatomy, physiology, and endocrinology. Objective knowledge lay in the material body, animal behavior was to be approached from “the deterministic point of view of the physiologist, treating overt behaviour as the result or expression of physiological events which have been made obvious through experimental analysis” (Zuckerman, 1932, pp. xi–xii). For these reasons, Zuckerman was deeply mistrustful ethology. He interpreted the observational methodology of ethology as retrogressive, threatening to reintroduce anecdote and anthropomorphism into behavioral science. Zuckerman held fast to this view throughout his career. Late in retirement, he reflected that Niko Tinbergen had been right to resist Julian Huxley's urging to consider the subjective elements of animal behavior, for this “would have taken Tinbergen back to the kind of anthropomorphic animal behavior studies that were so common in the nineteenth century” (Zuckerman, 1992, p. 162). These epistemological commitments help to explain why Zuckerman framed the minedog as an automaton.

In conceiving the dog as an automaton, Zuckerman denied the animal key characteristics of subjectivity: (1) intention in the form of authorship of action; (2) understanding of action as meaningful to the actor; and (3) temporal continuity of action (cf. Crist, 2000, p. 202). In doing so, Zuckerman significantly limited the ways in which his investigation could imagine and work with minedogs. For instance, by denying temporal continuity, and thus historical biography, it made no sense to ask how minedogs understood new experiences in view of the old. Accordingly, Zuckerman did not consider how prior experience (or memory) might shape present behavior and thus potentially explain the inconsistencies that beleaguered his investigation. This approach was partly necessary, in that it legitimated the basic experimental practice of arduous regimes of trials, retrials, training, and retraining. Zuckerman's experimental designs were premised on extensive repetition, with dogs trained for one task on one model environment, before being retrained for different trials on the wells, tunnels, or model minefields.^42^ The tedious and repetitious nature of the experimental work also challenged the human handlers and researchers. Ashton and Moulton complained that what was expected of them, though “theoretically admirable,” was both “technically difficult” and “beset with pitfalls” due to the basic method of repeating a given trial with interminable major and minor modifications.^43^ This approach not only assumed minedogs to be automatons but required them to be so. Had dogs possessed biographic history, then any given stimuli would appear within a context, memory would intervene as a variable. Consequently, dogs had to be denied a past. Framed as automatons, dogs operated as blank slates, responding to the immediate material stimuli of the environment alone, without reference to experience and historical interpretation.

Excluding subjectivity required Zuckerman to dismiss anecdotal knowledge and anthropomorphism, both being narrative forms that sustained subjecthood. This approach reflected what Eileen Crist has termed “mechanomorphism.” Counterpoised to anthropomorphism, mechanomorphism works to *extinguish* animal authorship by locating agency in “causes that are theoretically identified – for example neurophysiological mechanisms, environmental stimuli, or genetic programs” (Crist, 2000, p. 203). As a result, Zuckerman's investigation was epistemologically incompatible with prior approaches to, and understandings of, minedogs developed during the Second World War. Anecdotal knowledge and anthropomorphic interpretation were critical narrative forms sustaining earlier military practices. Richardson, for instance, warned against regarding “the dog too much as a machine” as this would undermine the “confidence and affection [which] must exist between dogs and keeper” (Richardson, 1920, pp. 64–65). For Richardson, and military training practices generally, the subjective state of a dog was an essential consideration. Training practices were built about productively managing the intersubjective relationships. In contrast, Zuckerman did not consider “confidence,” “affect,” or “love,” to be useful categories, either in the scientific investigation of minedogs or their practical usage (cf. Richardson 1923). One could not ask a man to wager his life on the “love” of a dog. Consequently, Zuckerman rejected on principle the established military approaches to working with dogs, refusing to engage with Richardson's principles or the tradition of animal training he represented. Across the Atlantic, however, a contemporous American investigation was underway that took a very different approach more amenable to working with the dog as a subject.

## Joseph B. Rhine: Working with Minedogs in a Shared World

In late 1951, as Zuckerman's enquiry was finally getting underway, the U.S. Army Engineer Research and Development Laboratories (Fort Belvoir, Virginia) initiated its own investigations.^44^ The American team, consisting of parapsychologist Joseph B. Rhine, anatomist Joseph E. Markee (both of Duke University, North Carolina), and biochemist Luman F. Ney (Stanford Research Institute, California), were immediately acquainted through military channels with the British work. Premised on the capacity of dogs to detect landmines through extrasensory perception (ESP), Rhine's investigation attempted to integrate anecdotal knowledge and anthropomorphic interpretation with experimental science.^45^ In April 1952, Zuckerman visited the Americans, returning with a resoundingly positive view of Rhine's parapsychological experiments. Despite Rhine's preference for nonphysical explanations, Zuckerman admitted that he had learnt much from him about “how to avoid the pitfall of self-deception, and how strict … controls have to be.”^46^ Rhine's impressive experimental designs, displaying sophisticated controls and highly developed statistical analysis, were in part inspired by the ephemeral nature of parapsychology. Widespread scientific skepticism about the reality of parapsychology ensured that those who studied it strove hard to assure their work met with expectations of experimental validity. Indeed, parapsychologists were often innovators, developing new tools and techniques in the pursuit of scientific objectivity (Mauskopf & McVaugh, 1980; Hacking, 1988; Dehue, 1997).

**Figure 5:**
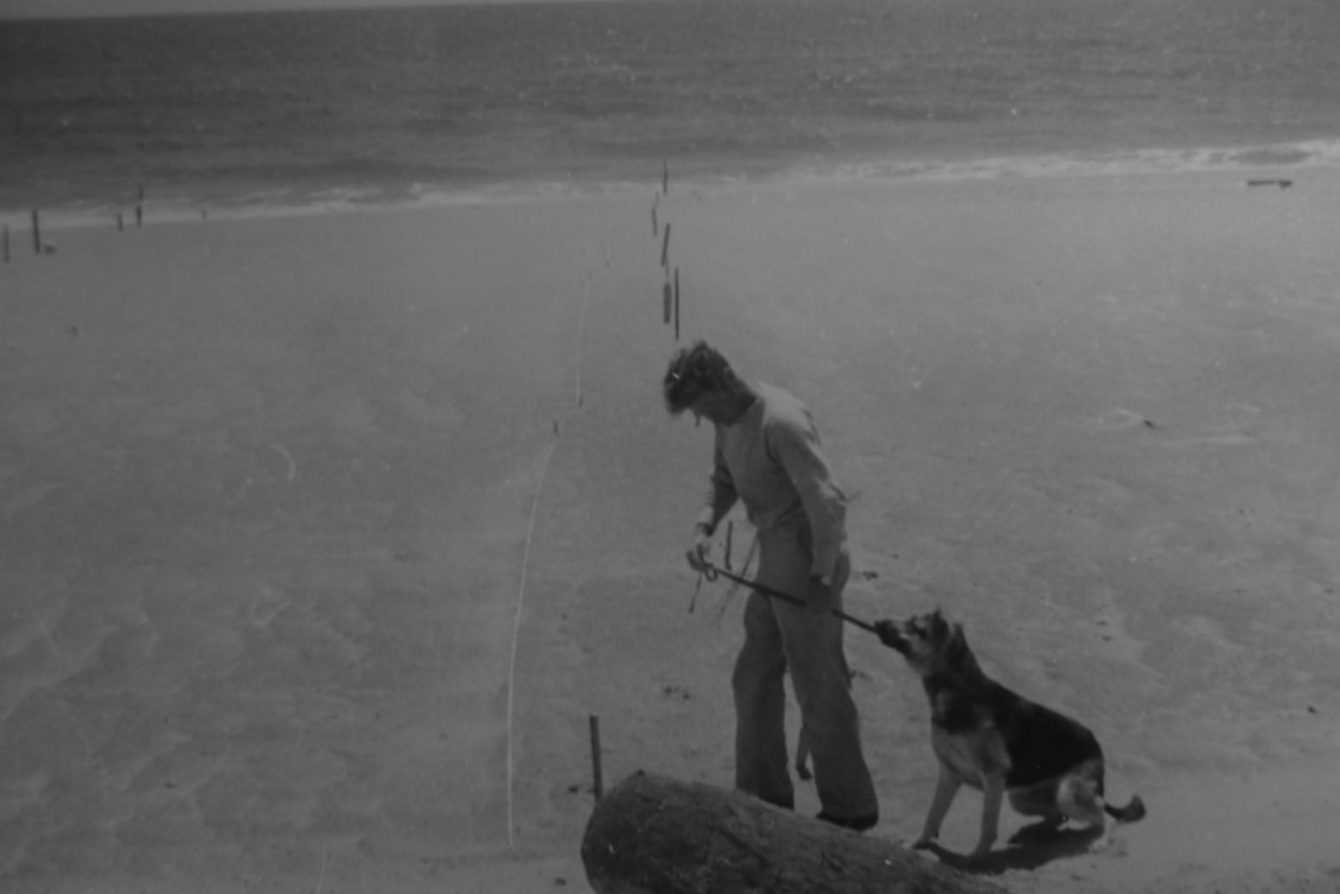
J. B. Rhine and minedog.* Note: *SZ CSA/188 Letter Leonard O Goff to S. Zuckerman, 9th July 1952, p. 7, ZA.

Like Zuckerman, Rhine designed his experiments around model environments, which he constructed on a sandy Californian beach. Landmines were placed in trenches that were subsequently filled, packed tightly, and raked so as to provide no tactile or visible cues. The experimental work was undertaken when the minefield lay beneath a shallow layer of seawater, which served to eradicate “normal” (physical) sensory cues (Figure 5).

To ensure that participants in the trials had no knowledge of the location of landmines, the model environments were constructed in the absence of members of the experimental team, including the dogs. Having been trained to sit to indicate a mine following standard military practice, trials involved a dog being led through the “minefield” by a handler. Results were collected by a designated observer. Delineating tasks in this way served to limit access to information thereby guarding against the risk of unconscious communication between researchers, handlers, and dogs. Rhine's investigation quickly established that dogs exhibited an initially high level of ability to detect landmines, followed by a slow decline to the point that success rates fell below that expected by chance (Rhine, 1971, p. 26). High initial success in a task followed by sharp decline was the established, albeit unexplained, hallmark of ESP (Rhine, 1934). All dogs consistently adhered to this pattern, convincing Rhine that they were employing ESP.

Making practical use of this ability would require understanding and overcoming the ESP “decline effect.” This, Rhine explained in a 1953 grant renewal request to the U.S. Army, would require additional support over “a matter of some years.”^47^ Rhine was hopeful the military would continue to support his work due to the wider “importance this factor would be to a military power who first acquired understanding of it.”^72^ However, officials at the Army Engineer Research and Development Laboratories (Fort Belvoir, Virginia) were less enthusiastic, shifting their attention and support to others who promised an objective “physical” (i.e., mechanical) approach to mine detection. Consequently, Rhine's funding ceased in 1953. In the work of mine detection, mysterious “decline effects” were deemed a serious defect.^49^ Rhine, like Zuckerman, had failed to produce a practically useful minedog. Yet, contrary to Zuckerman, Rhine had concluded that dogs could detect buried landmines and could prove useful in the field. The two investigations reached different interpretations of their failure due to their distinct epistemological commitments, which produced different approaches to incorporating human and animal subjectivity. Both had identified intersubjectivity as the site at which success or failure was decided. Indeed, this was the area where Zuckerman learnt most from Rhine, borrowing the latter's sophisticated precautions against unconscious communication. Nevertheless, their positions on the value of intersubjectivity were quite different. For Zuckerman, subjectivity could not be explained or reliably managed by the science of his day. Consequently, the scientific study of behavior had to frame animals as experiencing “only objective existence” (Zuckerman, 1932, p. 17). Zuckerman, therefore, implemented Rhine's techniques so as to erase subjectivity from his experimental designs. In contrast, Rhine saw subjectivity as a positive resource. Within parapsychological epistemology, subjectivity had to be properly managed if experimental science was to harness ESP. These epistemological orientations toward subjectivity had profound implications as they framed the permitted relations between human and minedog. In the course of their experimental investigations, it was this initial framing which, more than anything else shaped Zuckerman and Rhine's divergent conclusions.

## Relating to the Dog as a Subject

Rhine was compelled to include subjectivity within his science because parapsychological capacities, such as ESP, telepathy, and precognition, necessitated, by their very definition, a knowing subject (Rhine, 1937). Anthropomorphism was consequently of importance to Rhine as it provided a narrative form within which subjectivity could supervene. Anthropomorphism, in other words, allows the animal world to be represented as “a place of knowledge, emotion, intention, thinking and memory” (Crist, 2000, p. 202). For related reasons, Rhine also integrated anecdotal knowledge within his science. Anecdote, Rhine believed, was a valid form of knowledge that allowed access to the extreme variations in nature, the *paranormal* as opposed to the *normal*. Unlike Zuckerman, who placed anecdotal and experimental knowledge in opposition, Rhine located the two on a continuum:

the idea of using dogs to locate mines grew out of the same order of casual observation and collection of anecdotal material that we are now pursuing … In looking for the extremes that nature produces only occasionally do we hope to find the effect which eventually can be produced under experimental control.^50^

Anecdotal accounts of extraordinary animal behavior provided important starting points that Rhine hoped would guide parapsychology toward an animal model for ESP. Parapsychology's credibility as a science rested on gaining the same tools and methodologies as the biological sciences (cf. Mauskopf & McVaugh, 1980). An animal model for ESP, therefore, would build a bridge between parapsychology and the mainstream biological and behavioral sciences. For instance, Rhine believed that establishing ESP in animals would provide an evolutionary explanation for paranormal ability, opening up the possibility of identifying a “genetic foundation” for phenomenon such as clairvoyance and telepathy (Rhine, 1950).

Rhine's starting point in his search for credible evidence of nonhuman ESP (or “animal psi”) was to systematically identify, document, and investigate reports of extraordinary animal behavior. The most frequent example of such behavior involved cases of “trailing,” where abandoned animals tracked their owners across hundreds of miles without experience of the territory they crossed or their final destination.^51^ Improbable accounts of animal trailing were a common feature of American popular culture as population movement across the vast American landscape had long provided ample opportunity for the phenomenon. As a result of the geographic movement caused by the mobilization of American men during the Second World War, tales of animal trailing reached a height of popularity. American newspapers delighted in tales of heroic soldiers setting out to serve their country, only to be followed by their equally loyal dogs setting out to find their suddenly absent masters. Rhine collected every story and carefully investigated the most promising. In 1944, for instance, a black spaniel named Joker came to national prominence having traveled 6,000 miles from his Californian home to a South Pacific Island where he successfully located his “master,” Captain Stanley C. Raye (Anonymous, 1958). Cases such as this, Rhine believed, would help to establish the reality of animal psi, thereby initiating a revolution in biology as “animal instincts” were gradually shown to be paranormal mechanisms. Trailing was well established in the anecdotal animal behavior literature, Richardson, for instance, devoted a whole chapter to the “homing instinct” in dogs (Richardson, 1920, pp. 163–182). For Rhine, establishing that animals navigated through a sixth sense would make parapsychological considerations necessary across the behavioral sciences, opening the way to the study of more improbable claims, such as precognition (an area where again dogs predominated).

With a wealth of anecdotal trailing reports, Rhine chose for systematic investigation only those that had no likely natural explanation. Credibility rested on conclusively establishing the identity of the animal in question. Accordingly, comprehensive biographic histories were established for each animal, which correlated material evidence with witness testimonies. Collars, photos, and veterinary records were examined, anatomical characteristics noted, particularly distinguishing injuries such as scars from accidents or surgical interventions documented. Mannerisms, likes and dislikes, and other behavioral propensities were also logged and verified. In the case of Joker, for instance, witness testimony at each stage of the story established that the dog answered to this name. Indeed, Joker's journey was documented from so many independent sources that Rhine considered it “definitely one of the best cases of trailing we have.”^52^ The veracity of trailing phenomena rested on the accurate identification of an individual animal, necessarily assuming biography and animal subjectivity in the form of mindful intent. Yet, Rhine was not aiming to transform anecdotal into scientific knowledge. On the contrary, Rhine understood anecdote to be a type of knowledge *different from* but *equivalent to* experimental knowledge. Anecdote can be understood as an example of “case based reasoning,” a form of knowledge capable of producing generalized truths while sustaining the specificity, locality, and variety of its objects (cf. Forrester, 1996, 2007). Here, the anecdotal “case” fulfilled a comparable discursive role as the model in experimental knowledge (cf. Creager, Lunbeck, & Norton Wise, 2007). For Rhine, anecdotal knowledge was not only compatible with experimental science but a viable alternative for investigating phenomena that could not be captured experimentally. It could, therefore, stand in the stead of experimental knowledge (Rhine, 1950, p. 86). This was particularly important where it was impossible to construct a valid experiment, such as with trailing (Rhine & Feather, 1962).

**Figure 6:**
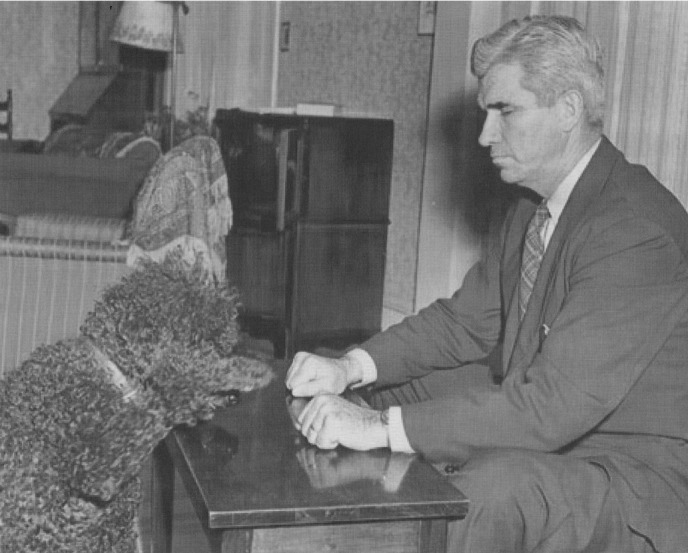
Publicity photo of Joseph B. Rhine testing a dog for ESP.

Common to all trailing cases was the breaking of a longstanding, strongly felt, bond, shared by owner and animal. This, Rhine believed, partially explained why the majority of cases involved dogs. As a species, dogs were characterized by “loyalty.” When a strongly felt bond was broken between owner and animal, the latter was able to “unlock” an otherwise “dormant” extrasensory ability in order to reassert their lost relationship (Rhine, 1950, p. 88). Due to the centrality of the intersubjective bond, testing this hypothesis experimentally proved impossible. The abandonment of a much loved animal, hundreds of miles from their owners, in the hope they might unlock latent paranormal abilities and return was unfeasible. The authenticity of the intersubjective bond negated the possibility of the act, for if one valued the relationship how could one knowingly place it at risk? Building and shattering bonds for experimental purposes was equally problematic. How could one verify whether a bond formed for instrumental purpose by one party would be sufficiently authentic to trigger ESP in the other? Attempts to modify “abandonment” so as to reduce risk were unsuccessful. For example, Rhine experimented with tethering pet dogs on long leashes before simulating abandonment in a forest. The leash was long enough to allow dogs to orientate themselves before setting off in the direction of “home” while protecting the dog from loss. Unfortunately, Rhine found dogs would refuse to accept the reality of their abandonment. Rather than heading in the direction of their owners at home, dogs instead sought out the concealed researchers tasked with observing the dog's choice of direction. Trailing, Rhine concluded, could not be investigated experimentally. Consequently, it could only be known and verified through anecdotal knowledge (Rhine & Feather, 1962). Rhine supported his trailing investigations with funds from the minedog contract, portraying anecdote as a potentially viable route to understanding and unlocking animal ESP compatible with experimental investigation. Similarly, funds were diverted to support the work of Karl Otis on ESP in cats (1953), and also helped to initiate Joseph Gaither Pratt's investigations of ESP in pigeons (Pratt, 1953).^53^ Together, these various approaches to the study of paranormal activity in animals illustrate how subjectivity formed an essential category within parapsychology (Figure 6). For these reasons, among others, Rhine's investigation could not be framed about the erasure of the dog as subject.

Historically, parapsychology coemerged with the study of animal mind (Lachapelle & Healey, 2010).^54^ Not least among their shared concerns was the methodological challenge posed to objective knowledge claims by the intersubjective relationship between observer and observed. Rhine had learnt of the potential for intersubjectivity to mislead in one of his earliest parapsychological investigations. In 1928, accompanied by his mentor William McDougall, Rhine was invited to attest to the paranormal abilities of Lady Wonder, a “mind reading” horse. Though initially convinced that this was an authentic case of cross-species telepathy, Rhine later concluded that Lady Wonder was actually responding to subtle unconscious physical cues (Rhine, 1929a, 1929b). The problem of unconscious communication, the so-called “Clever Hans effect,” was particularly challenging for parapsychologists because communication was not restricted to subtle physical cues. The paranormal, by definition, was predicated on the possibility of immaterial communication, for instance telepathy. Hence, in spite of all Rhine's methodologically sophisticated controls against unconscious communication, there necessarily remained the possibility of unconscious paranormal communication. Rhine could not exclude intersubjectivity because this was the vector by which paranormal activity occurred. Within parapsychology, therefore, monitoring and managing intersubjective relationships was central to experimental practice.

Zuckerman, of course, had no such epistemological limitation, and so was free to portray intersubjectivity as an entirely negative phenomenon. Indeed, intersubjectivity, which was interrelated with unconscious physical communication, became the main explanation for the inconsistencies in the behavior that plagued his investigation. Erratic minedog performance was ascribed to the fact “[s]ome dogs will work for one man and not another,” which conflicted with Zuckerman's regime of interchanging handlers and dogs. Early in the investigation Ashton reported that “the influence of the handler on the dog's performance is particularly marked,” which suggested that “sympathy between dog and handler” might be the condition of a productive working relationship.^55^ Zuckerman disagreed. The need for sympathy was apparent not real. Intersubjective factors such as sympathy was nothing more than a troublesome “biasing factor” that when removed would be found superflouous to successfully working with minedogs.^56^ However, erasing intersubjective relations proved an impossible task. Zuckerman's main technique was to rotate minedogs between different handlers in an effort to curtail familiarity and thereby block the formation of intersubjective relationships. When rotation failed to improve performance, or decrease behavioral inconsistency, the practice was intensified until a collapse in success rates required its abandonment. As a temporary compromise, to be overcome in the long term, rotation was restricted to the cycling of two dogs that were permanently attached to a handler. This system allowed for a “greater keenness on the part of the handlers and an improvement in the dogs performance.”^57^ Other techniques were introduced to disrupt intersubjective relations, none successfully. The most unusual, based on the principle that visual recognition was an integral component of building intersubjective relations, involved the introduction of blindfolds worn by the handler, dog, or both. Unsurprisingly, this had a disastrous effect on success rates and could never have been employed in the field. Zuckerman proved unable to overcome this fundamental tension between expectation and experience. His desire to erase intersubjectivity, both to ensure scientific objectivity and to produce a reliable minedog, could not be reconciled with the apparent instrumental necessity of cross-species intersubjective relations. Ultimately, this irreconcilable tension was the outcome of Zuckerman's interpretation, and subsequent epistemological position, on intersubjectivity and the “Clever Hans” effect. For Zuckerman, these were impure, uncontrollable, supplements, serving only to disrupt experimental science. They had to be excluded if reliable knowledge was to be produced.

Yet, as Vinciane Despret has shown, there are multiple ways of reading the Clever Hans story, each with distinct outcomes and ethical consequence. Despret suggests that a nonhuman animal's capacity to communicate with humans could be understood more positively and thus productively. From this perspective, Clever Hans's ability becomes a testament to his “capacity to be engaged” and “to align his action with what was expected of him,” albeit on the condition that human participants were equally willing to be “deeply engaged and interested” (Despret, 2004, p. 116). Rhine comes close to this approach, through his necessary conviction that intersubjective relationships were to be productively managed, not erased, if paranormal abilities such as ESP were to flourish. For Rhine, the intersubjective relationship was fragile as it served as the vector for paranormal activity. Any interventions had to be made with care. For instance, Rhine was aware that he had not controlled against the possibility that a handler might unknowingly locate a landmine through ESP and unconsciously communicate this knowledge to the dog through telepathy or other means.^58^ It would have relatively simple to ascertain whether handlers could independently detect mines via ESP by trialing them without the dogs. Yet, Rhine refused to do so. He feared that this “might have endangered the already delicate psychology of the handler to have him compete with his dogs,” which could only “undermine the dogs” (Rhine, 1971, p. 27).^59^ Instead, Rhine chose to place trust in the handler-minedog relationship.

Within Rhine's investigation, therefore, it ceased to matter which individual was responsible for detecting a mine. Increasingly, Rhine understood his task to be the creation of a mine-detecting “team” at the heart of which was the intersubjective relationship, quite the opposite of Zuckerman's aim of making the minedog into a transferable tool. Consequently, when Rhine explained his failure to produce a consistently reliable minedog team, he could speculate that “some needed factor gave out” that disrupted the cross-species intersubjective relationship. In one example, Rhine explained that the handler had become:

personally not himself. He was manifestly nervous, slightly irritable with his dogs, and, judging by little signs one learns to look for, not enthusiastic about going on with the project … he was frustrated and was dealing with something that was beyond him.^60^

Intersubjective relationships thereby provided a flexible explanatory framework for the successes and failures of Rhine's work. They also suggested a way forward, being the need to properly manage intersubjective relations so as to facilitate cross-species collaboration. When the minedog question resurfaced again in the context of Vietnam, Rhine was therefore able to reflect on his past work and conclude:

what stands out most emphatically today is … the question of whether it was perhaps the team and not either man or dog alone that produced such results (Rhine, 1971, p. 19).

Rhine went on to describe the ideal qualities of such a team, the most important of which being that “a functional integration of man and dog mentalities existed” (Rhine, 1971, p. 31). A decade later, despite the cognitive revolution having reintroduced the mind as a credible focus of study for the behavioral sciences, Zuckerman remained committed to his claim that that dogs were of no use as mine detectors.^61^

Zuckerman's interpretation of intersubjectivity reflects the conventional legacy of the Clever Hans story, that experimental practice be orientated about reducing the range of possible behavioral stimuli. Yet this also serves to impoverish the range of explanations for behavior (Despret, 2004). Rhine, as a result of the epistemological commitments of parapsychology together with the need to work with intersubjectivity, was much less constrained in how he related to, understood, and worked with nonhuman animals.

## Conclusion: Cross-Species Collaboration and Response-Able Relationships

Minedogs continue to divide opinion. Some within the mine clearance community, such as the HALO Trust, reject minedogs as an unproven technology. Others, for instance the Mine Advisory Group (MAG), have made use of Mine Detection Dogs for many years across Cambodia, Iraq, and Sri Lanka. Common to all those who subscribe to minedogs is the prioritization of intersubjective relationships. In the early twenty-first century, mine clearance with dogs is imagined as teamwork; consisting of human and dog within “a unit with equal and complementary roles” (GICHD, 2004, p. 29). A dynamic, flexible, and responsive approach to collaborative cross-species labor has superseded older notions of behaviorist-inspired control and obedience. Training is framed as an “interactive” process, designed to encourage “socialization” while imbuing the dog with “intent” and “motivation” for the work. Mine clearance is anthropomorphically understood from the point of view of the dog as a “game” in which the dog's “aim” is “to manipulate the handler to provide a desired object, such as a toy” (GICHD, 2004, p. 32). Central to the training process is attention to, and management of, cross-species intersubjective relationships. For instance, the “reward” is not just a material gain for the dog; it is intended to enhance the intersubjective bond. Reward giving is accompanied by positive gestures, including a “happy voice” and pleasurable body language such as petting, designed to express enthusiasm and thereby maintain the dog's “interest” in the “game” (McClean, 2003). Throughout, the language employed ascribes cognitive qualities to dogs, including “assessment,” “prediction,” “decision making,” and “judging.” Dogs are also credited with moral characteristics, such as “courage” defined as “understanding the danger of the minefield and accepting the risks involved” (GICHD, 2001, p. 31). In sum, twenty-first-century minedogs are ascribed subjecthood, they are presumed to be mindful authors of their own action, to possess personal biography. Accordingly, dogs are framed as distinct, but equal partners, helping to sustain the intersubjective relationship that is understood to be the key to successful cross-species collaboration.

The suggestion that intersubjectivity is a necessary condition for productively working with minedogs is, of course, concordant with Zuckerman's belief that minedogs were of no practical utility (given that he refused to work with the dog as a subject). However, orientations toward intersubjectivity have more than instrumental consequence. Smuts has described how intersubjective relations, even when involving a nonhuman animal, necessarily imply “the presence in another of something resembling a human self” in the form of “something we feel rather than something we know” (Smuts, 2001, p. 308). Moreover, as intersubjectivity assumes a relation with “someone so like ourselves that we can co-create a shared reality as equals,” it brings with it an obligation, if not a responsibility, to “care” (Smuts, 2001, p. 308). Rhine, for instance, demonstrated a sense of caring for his animals when he wrote that the older military training methods, that conditioned dogs using punishment, had “happily been abandoned” (Rhine, 1971, p. 22). We might ask, therefore, whether the refusal of intersubjectivity would lead to an absence of care and if so to what consequence?

Any approach to such a question from a historical perspective risks the error of mistakenly ascribing the moral sentiments and ethical principles of the present to the past. We can only understand the past on its own terms, by the moral and ethical standards of its time. Nevertheless, at all points in history, moral sentiments and ethical principles have been multiple. From this perspective, Zuckerman's investigation does display an absence of care derived from his refusal to countenance nonhuman subjecthood. In sourcing dogs, Zuckerman followed established military practice by obtaining stray animals from pounds, most often Battersea Dogs Home and the Birmingham City Pound (Anonymous, 1950; Zuckerman, 1988, p. 151).^62^ However, Zuckerman's intent was not just to *create* minedogs (as had the military) but to investigate their capacities *experimentally*. Put another way, Zuckerman used his dogs as experimental animals. In Britain, at the time, the sourcing of animals for scientific use was lightly regulated. Indeed, the only legal requirement appeared in Section 3(5) of the Dogs Act (1906), forbidding the release of stray dogs for the “purpose of vivisection.” Arguably, despite Zuckerman's experimental intent, as he planned no invasive surgery, his work could not be properly defined as “vivisection.” Despite vivisection having a much wider connotation in lay language, where it was commonly used to mean all forms of animal experimentation, Zuckerman did not consider his work to be “experiments” in the legal sense. In 1954, writing to his local Inspector, Zuckerman explained that his investigation did not meet the statutory definition of “experiment” as it was not calculated to cause pain; consequently, the work did not fall under the remit of the Cruelty to Animals Act (1876).^63^ Zuckerman was not being disingenuous. Conventionally, as a legacy of the historical context in which the Cruelty to Animals Act (1876) had been written, pain had predominantly been conceived physiologically by those who administered the law. As no invasive surgery was planned, there was no risk of physiological pain and so his work circumvented formal regulation.

Few within the regulatory community would have disputed Zuckerman's assessment, even had they known the extent to which his work caused “neurosis” in the dogs. Writing to Air Vice-Marshal T. McClurkin in 1952, Zuckerman explained that:

at this high level of work and training [t]he larger proportion of dogs is failing … Some of the animals literally had nervous breakdowns.^64^

Of 24 dogs trained that year, seven “proved unsatisfactory during training or retraining and were discarded,” while an eighth “promising worker” had become “unaccountably savage and had to be destroyed.”^65^ Zuckerman saw no relevance in these observations beyond the consequent need to maintain a regular supply of new dogs.^66^ Zuckerman displayed no interest in the frequently reported cases of dog neurosis, beyond their pragmatic and economic implications of increased turnover in dogs. Nor did he feel the need to conceal the phenomenon. Neurosis simply had no relevance to his investigation. Within the context of the time, there is nothing exceptional in Zuckerman's lack of interest in neurosis as it was a well known by-product of various techniques commonly used across the behavioral sciences. Researchers had grown used to discarding animals as unusable should neurosis occur. Relatively few altered their interests to make “experimental neurosis” the object of their research (notable exceptions included Pavlov, Gantt, and Liddell).^67^ Even so, there was little if any debate as to whether animal neurosis constituted suffering. In Britain, as the Cruelty to Animals Act (1876) applied only to experimental practices that were “calculated to give pain,” it relied on scientists accurately assessing whether or not their proposed work met this condition and acting accordingly. Statutory definitions of pain and suffering, therefore, relied on scientists themselves reporting and explaining why they believed their proposed work was “calculated to give pain.” During the 1950s, the statutory tendency to define pain physiologically was beginning to be questioned in response to evolving scientific understandings of new concepts such as stress (Kirk, 2014). Mental suffering, however, could not be attributed to animals when the accepted wisdom of the scientific community refused to attribute mind to animals. In the 1950s, therefore, those who equated animal neurosis with mental suffering were initiating a sea change in established understandings of animal experience that would take decades to supplant established beliefs (e.g., Croft, 1951; c.f. Kirk, 2009).

While representative of the period, by excluding internal subjective experiences, Zuckerman severely limited the questions he could ask of dogs. Not only did he close down the possibility of adequately caring for a dog's mental state, Zuckerman limited the extent to which mental suffering may have shaped the behavioral inconsistencies that dogged his investigation. Zuckerman could not ask, for instance, how the effect of past experience upon future expectations might shape a dog's behavior. In part, the absence of personal history was an experimental necessity as it legitimated the central practice of training and retraining dogs for trials across the various model environments. Zuckerman's failure to adequately imagine the mental experience of minedogs produced an experimental design wherein animals were effectively presented with “conflict scenarios.” Placing animals in a position where they learnt that one set of actions led to a certain reward, only to have that certainty challenged once successfully acquired was strikingly similar to techniques commonly used for the deliberate production of experimental neurosis. Zuckerman was not ignorant of this connection; he witnessed a directly comparable proposition in 1952 when visiting Rhine's investigation. On a day when the American trials had embarrassingly fallen into disarray, Zuckerman recorded that:

Part of the failing of the training was attributed to … having started out to train dogs to detect buried mines, it was suddenly decided to train the dogs to respond to empty holes. This, it was thought, had occasioned a slight “neurosis” (in the dogs).^68^

Perhaps due to his antipathy for even a suggestion of subjectivity, Zuckerman neglected to translate this experience to his own investigation. Neurosis, after all, was a product of the personal history of an animal. Furthermore, the study of experimental neurosis often led investigators into the realms of intersubjectivity. W. Horsley Gantt, for instance, believed that “experimental neurosis” in dogs could only be understood if one studied the animal over an extensive period of time (Gantt, 1944). By doing so, Gantt developed his theory of the “effect of person,” which attempted to map intersubjectivity by identifying a dog's physiological responses to the presence and absence of others (Gantt et al., 1966).

Zuckerman's exclusion of internal mental life also precluded the possibility of explaining behavioral inconsistencies through the concept of *boredom*. Zuckerman was conscious of the fact that his experimental designs, grounded as they were on testing minor variations through extensive repetition, made “the work tedious and monotonous.”^69^ Again, however, the suggestion that animals could experience boredom was not of this time. Heidegger, for example, who drew heavily upon the biological sciences of the 1920s and 1930s to define his understanding of human experience (*Dasein*), distinguished human from nonhuman on the basis of the former alone possessing the capacity to experience boredom (Krell, 1992; Agamben, 2004). Significantly, twenty-first-century minedog discourse invokes “boredom” as an explanation for performative inconsistency:

A dog that is relatively new to detection work may deliver very intensive and focused search behaviour because it is stimulated and excited by the new learning situation … [but] after more experience, the dog may become sluggish and appear bored (McLean, 2003, p. 28).

Here, the concept of boredom introduces a powerful level of flexibility to interpretations of minedog behavior. Ascribing animal boredom has also altered material practice by encouraging the managed introduction of variety to the dogs’ daily work environment (while the nonwork environment is standardized so as to be devoid of stimulation). This is the precise opposite of Zuckerman's approach. Contrary to its intent, Zuckerman's attempts to create uniform model environments, rather than working to reduce behavioral inconsistencies, may have been their cause. Notably, military practices developed during the Second World War encouraged dog handlers to consider boredom as a cause for a dogs’ loss of keenness. Handlers were warned to be attentive to the personal, subtle signs, which indicated when a dog had lost “interest,” and told to immediately rest the dogs when this occurred. General guidelines were also introduced, one example stipulated that dogs “should not be worked longer than a half hour at a stretch, and should be given 15–30 minute rest after each spell of duty.”^70^ Rhine, too, recognized and regretted the negative effects of the “long, tedious period of training and retraining the dogs.”^71^

Acknowledging boredom reintroduces the individual dog's subjective experience of their world. Furthermore, for a human to understand the diverse and subtle ways in which a dog might communicate their state of being requires a dynamic, responsive relationship, built through personal experience of the other, what Donna Haraway has termed a “response-able” relationship (Haraway, 2008, p. 71). Relating response-ably requires participants to be attentive to the “inside connections that demand and enable response” (Haraway, 2008, pp. 70–71). Such an orientation, which entails a constant and evolving *interest* in the changing other, necessarily has ethical consequence. As Haraway explains:

Just *who* is at home must permanently be in question … one cannot *know* the other or the self, but must ask in respect for all time who and what are emerging in relationship … all ethical relating, within or between species, is knit from the silk-strong thread of ongoing alertness to otherness-in-relation. We are not one, and being depends on getting on together (Haraway, 2003, p. 50).

Haraway contrasts this approach to “bare calculation or ranking,” which alone can never be enough to facilitate productive cross-species labor (Haraway, 2007, p. 71). Here, Haraway is developing Despret's observations on the critical importance of “interest.” Despret advances a comparable contrast by way of asking which of two questions hold the most interest:

that of an animal strictly determined by its hormones and by hierarchical rules … or that of an animal articulating its body to other bodies, in a spirit of both competition and coordination to invent a solution to several problems. (Despret, 2005, p. 367).

For Despret, valuable science is transformative, a process of “becoming with” and “becoming together” (Despret, 2004, p. 122). Participants must be “activated as a subject both creating and created by passions,” thereby actively sharing in “an effort to become interested, to immerse oneself in the multitude of problems presented … to grow … to care” (Despret, 2004, p. 131). This was not the science of Zuckerman. However, it resonates with the practices of those who have come to believe that minedogs can be trusted in the minefield. The latter frequently display an openness to the transformative power of response-able relating.

We might conclude, therefore, by returning to Despret's observation that “learning how to address the creatures being studied is not the result of scientific theoretical understanding, it is the *condition* of this understanding” (Despret, 2004, p. 131). This observation, when taken seriously, invites those who study animals in science to move beyond the question of how animals are muted by scientific practices, such as docility (Arluke, 1994), transformation (Lynch, 1988), or standardization (Clause, 1993; Kohler, 1994; Rader, 2004). Instead, we might seek to understand how animals have been, and may yet be, given the opportunity to collaborate in human labor without their reduction and without the need to erase their subjecthood. This is particularly important when it is recognized that scientific practice, knowledge claims, and ethical responsibility are historically coemergent. The work that can be achieved with animals has never been isolated from the ways in which we relate to them, or the ways in which we care for them. Moreover, when we ask questions of animals, we place at stake not only the work we can achieve together but also who we are, who we want to be, and who we might become (“we” being inclusive of the nonhuman animal).

## Footnotes

HeroRATS operate by scent and are said to be able to detect landmines as well as tuberculosis in human sputum. See http://www.apopo.org/.

WO 32/10800, War dogs: Provision of war dogs, 1942-5, National Archives, Kew, UK (hereafter NA).

WO 195/9544, Physics of Armaments Committee: Land mine detection, November 5, 1947, p. 4, NA.

WO 291/1048, The detection of mines by dogs, Interim report, c.1947, p. 2, NA.

Americans, in contrast, unsuccessfully experimented with conditioning through punishment; see WO 205/1173, Note on a visit to British war dog training centre March 13, 1945, p. 2, NA.

AVIA 24/27, Use of dogs for mine detection, June 3, 1944, NA.

WO 291/1048, On the detection of mines by dogs, April 1947, p. 2, NA.

AVIA 24/271, Mine detection dogs, August 11, 1944, p. 1, NA.

WO 204/4781, Mine field location by dogs, November 26, 1943, NA.

AVIA 24/271, Use of dogs for mine detection, June 3, 1944, p. 3, NA.

Retraining a dog to a new handler took two weeks, see WO 205/1173, Notes on visit to British war dog training centre on March 13, 1945, p. 2, NA.

WO 205/1173, Training instructions on the handling of dogs for mine detection, c. June 1944, p. 2, NA.

AVIA 22/871, Use of dogs for mine detection, June 3, 1944, NA.

AVIA 24.271, Detection of mines by dogs, August 6, 1944, p. 2, NA.

AVIA 24.271, Mine detection dogs, August 11, 1944, p. 2, NA.

AVIA 24.271, Report of employment of No. 1 dog between July 15 and August 11, 1944 in Normandy, p. 3, NA.

WO 291/1048, Detection of mines by dogs, Interim report, c.1947, p. 1, NA.

AVIA 24.271, Mine detection by dogs, undated c.1944, p. 1, NA.

AVIA 24.271, Report of employment of No. 1 dog between 15 July and 11 August 1944 in Normandy, p. 3, NA.

AVIA 24.271, Mine detection dogs, August 11, 1944, p. 2, NA.

WO 291/1048, Detection of mines by dogs, Interim report p. 2; see also AVIA 22/871 Detection of mines by dogs, NA.

SZ/CSA/188, Letter Colonel S. A Stewart (Royal Engineers Development, Ministry of Supply) to Solly Zuckerman, August 6, 1948, Zuckerman Archive, University of East Anglia, Norwich, UK (hereafter ZA).

WO 195/9544, Land mine detection, November 5, 1947, p. 4; Physics of Armaments Committee: Land mine detection, 1947, p. 5, NA.

SC/CSA/188, Minutes of the first meeting of the land mine detection subcommittee, August 27, 1948, p. 1, ZA.

Zuckerman's Tots and Quots dining club were the anonymous group behind the programmatic *Science in War* (Anonymous, 1940).

SZ/OEMU/42/1/23, Minefield casualties, 18/9/45, ZA.

SC/CSA/188, Letter Solly Zuckerman to O. H. Wansbrough-Jones (War Office), June 24, 1949. Zuckerman intentionally framed his experimental approach to animal psychology in opposition to the then emerging new field of ethology. He saw the observational approach of the latter as a step back toward anecdotal studies of animal behavior. In 1949, the only institution undertaking studiesof animal behavior in the experimental style of Zuckerman was the Institute of Experimental Psychology (Oxford, established 1947), which had adopted an empirical and experimental approach as a strategy to avoid hostile criticism from Oxford University's vociferous philosophers. SC/CSA/188, Letter Solly Zuckerman to O. H. Wansbrough-Jones (War Office), June 24, 1949, ZA.

In 1951, for example, Kurt Danziger, then a recent graduate of Oxford's Institute of Experimental Psychology and now a notable historian of psychology, refused the position believing it would curtail his career. See SC/CSA/188, Letter Solly Zuckerman to Air Vice Marshall T. McClurkin (Ministry of Supply), May 23, 1949; Letter J. T. Eayrs to Solly Zuckerman, March 12, 1951; Letter Solly Zuckerman to K. Danziger, March 17, 1951, ZA.

On the basis of this work, Moulton was to build a career dedicated to the study of the chemical senses (Moulton, 1981).

SC/CSA/188, Minutes of the first meeting of the land mine detection subcommittee, August 27, 1948, p. 2; Letter S. Zuckerman to Colonel S. A. Stewart, August 6, 1948, ZA.

SZ/CSA/188, Letter Solly Zuckerman to E. V. D. Glazier, February 7, 1953, ZA.

SZ/CSA/188, Letter Solly Zuckerman to Colonel S. A. Stewart (Royal Engineers Development), August 6, 1948, ZA.

SZ/CSA/188, Letter S. Zuckerman to E. D. Adrian, August 27, 1956, p. 3, ZA.

Ibid.

SZ/ CSA/188, Use of dogs in mine detection: Report of progress work at Melton Mowbray during the year ending November 1952, p. 6, ZA.

SZ/CSA/188, Interim report on the use of dogs for detecting mines, November 3, 1951, p. 5, ZA.

A comparable conflict appears in the work of Ivan Pavlov. In a compelling study of Pavlov's working practices, Todes reveals how a tension between dog-as-technology and dog-as-organism was rooted in the dog's “lifestyle” and the confrontation between its biological complexity and Pavlov's scientific vision (Todes, 2002, p. 94). As Pavlov's research transitioned from gastric physiology to the study of the nervous system, he became increasingly interested in interplay of external lifestyle and internal psyche. Zuckerman, in contrast, insisted that internal psychic states were excluded from his vision of experimental psychology.

SZ/CSA/188, Detection of mines by dogs: Report on progress of experimental work at Melton Mobray and Christchurch during the period December 1952 to April 1954, p. 9, ZA.

SZ/CSA/188, J. T. Eayrs, The detection of mines by dogs: An analysis of present knowledge and experimental approach, July 27, 1951, p. 4, ZA.

Ibid, p. 10; SZ/CSA/188, Detection of mines by dogs: Report on progress of experimental work at Melton Mowbray and Christchurch during the period December 1952 to April 1954, p. 10, ZA.

Russell continued “Nay, more, they have all displayed the national characteristics of the observer. Animals studied by Americans rush about frantically, with an incredible display of hustle and pep, and at last achieve the desired results by chance. Animals observed by Germans sit still and think, and at last evolved the situation out of their inner consciousness” (Russell, 1927, pp. 32–33).

SZ/CSA/188, Use of dogs in mine detection: Report of progress work at Melton Mowbray during the year ending November 1952, p. 8, NA.

Ibid., p. 9.

All records relating to contract DA-44-009 eng-1039 were returned to the Engineer Research and Development Laboratories (Fort Belvoir, Virginia) and destroyed in 1969 after declassification. Details relating to costs survive in Box 132, Administration Folder, The Records of the Parapsychology Laboratory, 1893–1984, Rare Book, Manuscript, and Special Collections Library, Duke University, North Carolina (hereafter RPL). A copy of the contract and a small amount of administrative correspondence relating to costs survive in the Joseph E. Markee Papers (hereafter JEM), Box 2, Folder 27, Medical Center Archives, Duke University, Durham, North Carolina. Material shared with the British and passed to Zuckerman, including a copy of the final report, survive in SZ/CSA/188, ZA.

Rhine's duplicates of Richardson's writings survive in RPL Accession 2009 0089 Box 8, 9, and 12, and Box 10 of the Louisa E. Rhine Papers, Rare Book, Manuscript, and Special Collections Library, Duke University, North Carolina.

SZ CSA/188, S. Zuckerman, Detection of mines by dogs American experiments, 1952, p. 7, ZA.

SZ CSA 188, Letter J. B. Rhine to Commanding Officer ERDL, Fort Belvoir (Final Report) July 10, 1953, p. 11, ZA.

SZ CSA 188, Letter Solly Zuckerman to T. McClurkin, November 30, 1953, ZA.

SZ CSA 188, Letter J. B. Rhine to Commanding Officer ERDL, Fort Belvoir (Final Report) July 10, 1953, p. 11, ZA.

Hundreds of anecdotal trailing reports survive in RPL Box 10, and RPL Accession 2009 0089 Box 8, 9, and 12.

Letter J. B. Rhine to Stanley C. Raye, February 6, 1952, PRL Accession 2009-0090, Box 12. Rhine learned how Raye, on receiving his departure orders, had left the dog in the care of a friend in Pittsburgh, California. Joker quickly became unsettled in the absence of his master, constantly moving around the house and refusing to rest, eventually disappearing on the November 8, 1944 (13 days after Raye's own departure). Two U.S. Army medical officers, James P. Martin and Robert Carver, testified to finding the dog stowed on their transport, the Bosfontein, which had left the Port of Oakland (45 miles from Pittsburgh) on November 12. When discovered, the dog was adopted by a Major on route to the same Pacific Island that Raye had been stationed. After arrival, while out walking his new pet, the unnamed Major was most surprised by Joker's enthusiastic reaction on sighting Reye. See Trailing Case, Raye—Joker, PRL Accession 2009-0090, Box 12.

Consequently, Rhine overspent the grant, much to the annoyance of the Dukes financial administrators, see JEM, Box 2, Folder 27, Letter Paul Gross (Vice President, Duke University) to J. B. Rhine February 7, 1952. Rhine's support of Pratt, however, proved lucrative in that it led to a decade of further funding by the Office of Naval Research, see Pratt files in RPL Box 524.

Rhine, for instance, frequently wrote for the popular press on animal mind (Rhine, 1951c, 1951d, 1955). This historical legacy helps to explain the close relationship between parapsychology and ethology. For instance, Alastair Hardy, Linacre Professor of Zoology and Comparative Anatomy at Oxford, befriended Rhine through a shared interest in parapsychology (Hardy, 1950). He also supported ethology, bringing Tinbergen to his department in Oxford. Similarly, Donald Griffin, who later controversially developed the field of cognitive ethology, studied pigeon homing in the 1950s in a parallel investigation to that of the Duke parapsychologist Joseph Gaither Pratt. Both were funded by the Office of Naval Research. Copies of Griffin's Research Reports, together with correspondence between Pratt and Griffin, survive in RPL Box 524.

SZ/CSA/188, The Detection of mines by dogs: An analysis of present knowledge and experimental approach, July 27, 1951, p. 9, ZA.

SZ/CSA/188/4, Detection of mines by dogs: Report of progress for period January to December 1955, January 1955, p. 4, ZA.

SZ/ CSA/188, Use of dogs in mine detection: Report of progress work at Melton Mowbray during the year ending November 1952, p. 2, ZA.

This general parapsychological problem was eventually resolved by completely automating the process of testing and observing precognition (Duval & Montredon, 1968).

Zuckerman, in contrast, tested human handlers, revealing that they could reliably find recently buried mines by sight (Zuckerman, 1988, p. 51).

SZ CSA 188, Letter J. B. Rhine to Commanding Officer ERDL, Fort Belvoir (Final Report) July 10, 1953, p. 10, ZA.

From the 1970s, the growth of cognitive ethology began to reframe the study of animal subjectivity so as to be a credible scientific activity (e.g., Griffin, 1976, 1992).

In 1953, finding his animals frequently of poor health, Zuckerman arranged for dogs to be supplied by Arlington Farms, Porton, a recently established breeding facility for the Ministry of Supply's Microbiological Research Department.

Zuckerman wrote “purely as a matter of form” to alert the Inspector to “secret work on mine dogs” that “does not concern experiments under the Act,” see SZ/CSA/188, Letter Solly Zuckerman to F. Elliot (Home Office Inspector), June 1, 1954, ZA.

SZ/CSA/188, Letter Solly Zuckerman to Air Vice-Marshal T. McClurkin, February 23, 1952, p. 2, ZA.

SZ/CSA/188, Use of dogs in mine detection: Report of progress work at Melton Mowbray during the year ending November 1952, p. 2. Once loss to disease were added to these figures wastage of dogs amounted to 50 percent.

SZ/CSA/188, Detection of mines by dogs: Report on progress of experimental work at Melton Mowbray and Christchurch during the period December 1952 to April 1954, p. 2 (Anonymous, 1950). Once loss to disease was added to the problem of neurosis dog wastage amounted to 50 percent.

See problems of neurotic behavior, p. 3, File Conference on Neurosis, Series II, Box 62, Robert Yerkes Papers, Yale Library Manuscripts and Archives, Sterling Memorial Library, New Haven; Pavlov (1932).

SZ/MOS/LMD/2, Detection of mines by dogs—American experiments, April 1952, p. 5, ZA.

SZ/CSA/188, J. T. Eayrs, The detection of mines by dogs: American analysis of present knowledge and experimental approach, July 27, 1951, p. 9. Describing progress in 1955 Zuckerman wrote of his research assistant “who is engaged full-time in this work … shivering on the job … burying mines along the sea-shore and following reluctant dogs as they refuse to find them!,” SZ/CSA/188, Letter Solly Zuckerman to E. D. Adrian, January 21, 1955.

WO 205/1173, Training instruction on the handling of dogs for mine detection, June 1944, p. 1, NA.

SZ CSA 188, Letter J. B. Rhine to Commanding Officer ERDL, Fort Belvoir (Final Report) July 10, 1953, p. 11, NA.

Idib.
